# Rehospitalization due to COVID-19 reinfection in Rio de Janeiro, 2020–2022: Associations with vaccination status, sociodemographic and clinical factors

**DOI:** 10.1371/journal.pone.0332281

**Published:** 2026-10-01

**Authors:** Ranna Kíssia Alves das Neves, Leonardo Soares Bastos, Daniel Antunes Maciel Villela, Valeria Saraceni, Débora Medeiros de Oliveira Cruz, Luiz Max Carvalho, Antonio Guilherme Pacheco

**Affiliations:** 1 National School of Public Health, Oswaldo Cruz Foundation, Rio de Janeiro, Brazil; 2 Scientific Computing Program, Oswaldo Cruz Foundation, Rio de Janeiro, Brazil; 3 School of Applied Mathematics, Getulio Vargas Foundation, Rio de Janeiro, Brazil; 4 Institute of Social Medicine/UERJ, Rio de Janeiro, Brazil; Independent Medical Researcher and Writer, UNITED KINGDOM OF GREAT BRITAIN AND NORTHERN IRELAND

## Abstract

The COVID-19 pandemic posed significant challenges, including rehospitalization of patients due to SARS-CoV-2 reinfection, particularly in the context of variants of concern and Brazil’s COVID-19 vaccination campaign. This study aimed to estimate the rate of rehospitalizations due to reinfection in the municipality of Rio de Janeiro (2020–2022), assess the impact of individual vaccination status, and identify factors associated with this outcome. Cohort study including individuals aged 18 years or older who were hospitalized with Severe Acute Respiratory Illness (SARI) resulting from SARS-CoV-2 infection in Rio de Janeiro. Data from four administrative databases (SIPNI, e-SUS Notifica, SIVEP-Gripe, and SIM) were integrated through record linkage. Rehospitalizations due to reinfection were defined as those occurring ≥90 days after the initial hospitalization. Annual rehospitalization rates were calculated per 100 person-years. The analysis included demographics, hospital discharge disposition, and vaccination-related variables. Cox proportional hazards models with deaths considered as competing risks were used. Of the 59,001 individuals included, 1,957 (3.3%) experienced rehospitalization. The main finding was that a complete vaccination regimen with a booster (≥3 doses) was associated with a 39% reduction in the risk of rehospitalization (sHR: 0.61; 95% CI: 0.52–0.73), while older age, multimorbidity, and Black race/skin color emerged as significant risk factors. Full vaccination with booster doses offered protection against COVID-19-related rehospitalization; however, racial inequities, advanced age, and comorbidities remain important risk factors. Public health policies that expand periodic booster vaccination, strengthen health education, and address social vulnerabilities are urgently needed as a fundamental strategy for preventing future pandemics.

## Introduction

The COVID-19 pandemic posed a major challenge to the Brazilian healthcare system, characterized by high incidence, widespread transmission, and elevated mortality. It demanded improvements in access to care, quality of services, surveillance, and control strategies [1]. In this complex scenario, the possibility of patient rehospitalization emerged as an ongoing concern [2], especially considering the circulation of viral variants of concern (VOCs), which raised questions about vaccine effectiveness and the potential for reinfection [3].

Brazil launched its national COVID-19 vaccination campaign in January 2021, initially with the inactivated virus vaccine CoronaVac and the viral vector vaccine ChAdOx1 nCoV-19/AstraZeneca. Later, the National Immunization Program (PNI) incorporated mRNA vaccines (Pfizer-BioNTech) and viral vector vaccines (Janssen – single dose). While mass vaccination led to a significant reduction in mortality and severe cases, shaping the course of the pandemic, other concurrent factors may have influenced clinical outcomes [4].

Recent studies indicate that Brazil accounted for a substantial proportion of COVID-19 cases in South America, in a context marked by significant regional inequalities in the availability of healthcare resources [5]. These structural limitations contributed to variations in case severity and clinical outcomes across different regions of the country. In the municipality of Rio de Janeiro, more than 1.2 million confirmed cases, nearly 20% of the population and 38 thousand deaths were recorded by December 2022, alongside more than 18 million vaccine doses administered [6]. In this context, there is limited evidence integrating linked clinical, epidemiological, and vaccination data to specifically examine rehospitalization due to reinfection, particularly in large urban centers, and it remains insufficiently explored to what extent different vaccination statuses, including booster doses, influence the risk of rehospitalization following reinfection.

According to the technical definition adopted in Brazil, COVID-19 reinfection was defined as a new symptomatic episode occurring at least 90 days after the first infection [7]. Monitoring COVID-19 rehospitalizations due to reinfection provides an important opportunity to assess vaccine-conferred protection beyond primary outcomes and to identify more vulnerable populations. This outcome is particularly relevant as it captures both disease severity and healthcare system burden over time.

Although the World Health Organization has declared the end of the Public Health Emergency of International Concern (PHEIC), it is important to continue monitoring COVID-19 due to the ongoing presence of the virus and its ability to cause severe illness, particularly in unvaccinated individuals and those with comorbidities [8].

In this context, the present study aims to estimate the annual rate of COVID-19 rehospitalizations due to reinfection in the municipality of Rio de Janeiro (2020–2022), assess the impact of individual vaccination status, and identify demographic and clinical factors associated with this outcome.

## Materials and methods

This study was reported in accordance with the STROBE (Strengthening the Reporting of Observational Studies in Epidemiology) guidelines, and the corresponding checklist is provided as S1 File.

### Study design, population, and data sources

This is a retrospective explanatory cohort study based on linked administrative databases, including individuals aged 18 years or older, residing in the municipality of Rio de Janeiro, who were hospitalized and reported with a diagnosis of Severe Acute Respiratory Illness (SARI) due to confirmed or unspecified SARS-CoV-2 infection, in both public and private healthcare sectors, between 2020 and 2022. The study included all eligible individuals identified in the linked databases; therefore, no a priori sample size calculation was performed.

The data were accessed in June 2024 and obtained from a database constructed through a previously validated record linkage process. Linkage was performed using an algorithm implemented in Python, comparing name, mother’s name and date of birth among records, as detailed in Pacheco (2008) [9]. After the procedure, all databases were anonymized and unique identifiers were attributed to records in the various databases that belong to the same individual. All analyzes were conducted on anonymized datasets.

This database integrated information from four administrative datasets related to the COVID-19 pandemic, provided by the Epidemiological Intelligence Center (CIE) of the municipality of Rio de Janeiro: 1) National Immunization Program Information System (SIPNI) – COVID-19 vaccination; 2) e-SUS Notifica; 3) Influenza Epidemiological Surveillance Information System (SIVEP-Gripe); 4) Mortality Information System (SIM).

To distinguish between rehospitalizations due to reinfection and those resulting from prolonged hospital stays or recurrence of the same infection, a 90-day interval between the date of first symptoms criterion was adopted, following the recommendations of the Brazilian Ministry of Health [7]. Thus, only hospitalizations with symptom onset ≥ 90 days after the first hospitalization were considered reinfections. This 90-day window was also used to control for immortal time bias, as events such as rehospitalization or death occurring during this period were not counted as time at risk [10]. Therefore, follow-up began only 91 days after the date of symptom onset of the first hospitalization (Fig 1).

**Fig 1:**
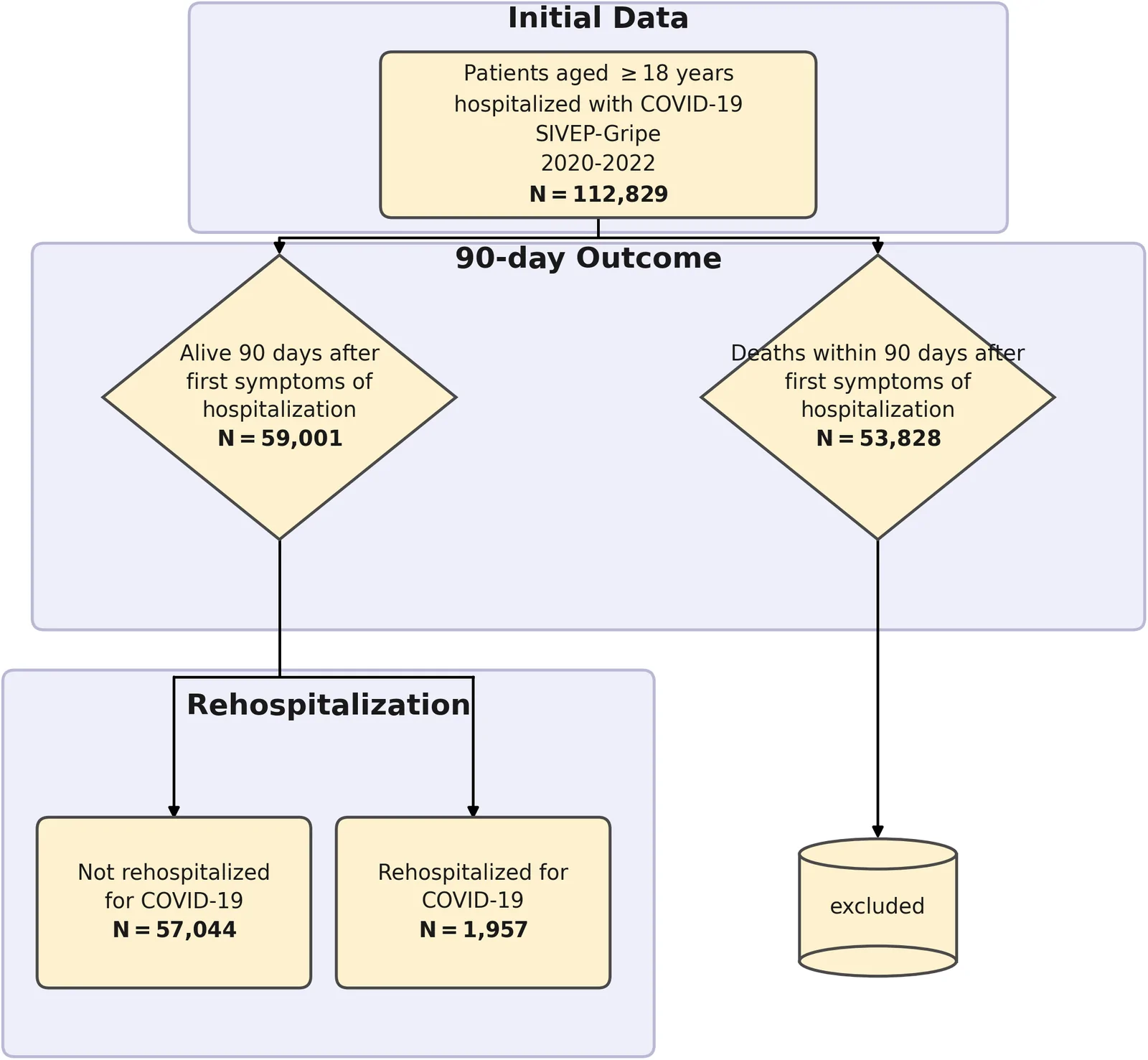
Flowchart of the selection of the cohort of hospitalized COVID-19 patients in Rio de Janeiro (2020–2022).

### Outcome and variables

The primary outcome was time to first rehospitalization due to COVID-19 reinfection. The study entry point was defined as the date of symptom onset for the initial hospitalization for COVID-19 or unspecified SARI, as recorded in the SIVEP-Gripe database. Follow-up started 91 days after this date and continued until the first occurrence of: (i) symptom onset for rehospitalization due to reinfection, (ii) death (treated as a competing event), or (iii) administrative censoring at the end of the study period (December 31, 2022), whichever occurred first. Deaths were treated as competing events in the analysis; adjustments were made for potential confounding factors.

The study evaluated multiple exposures, including demographic, clinical, vaccination-related, and contextual factors, in relation to the risk of rehospitalization due to COVID-19 reinfection. Demographic variables considered were age (in years), categorized age groups (18–49, 50–69, and ≥ 70 years), sex (male or female), and race/skin color was classified according to Brazilian Institute of Geography and Statistics (IBGE) categories as White; Black (including individuals self-identified as Black or Brown); and Others (including Yellow, Indigenous, or missing).

Regarding clinical characteristics, the presence and number of comorbidities (none, one, two, or three or more) were included, based on diseases recorded in the SIVEP-Gripe system. The comorbidities considered encompassed Down syndrome, cardiovascular, pulmonary, renal, hepatic, autoimmune, neurological, hematological diseases, diabetes, and others. Additionally, the final case classification was taken into account (SARI due to COVID-19, unspecified SARI, or missing), admission to the intensive care unit (ICU), categorized as yes, no, or missing and hospital discharge disposition (cure, death due to COVID-19, death due to other causes, or missing).

Vaccination status was classified into two categories according to the recommendations of the Brazilian Ministry of Health [11]: (1) complete vaccination with booster, defined as individuals who received three or more doses of any COVID-19 vaccines, except Janssen, for which two or more doses were considered as a boosted scheme; and (2) other statuses, encompassing unvaccinated individuals, those with incomplete vaccination schedules, or those who completed the primary series without a booster. Vaccination was considered effective starting 14 days after the administration of the last dose received.

An ecological variable was created to determine the dominant SARS-CoV-2 variant. This variable was based on the Epidemiological Bulletins from the municipality of Rio de Janeiro [6], which define the periods of viral variant predominance as follows: (pre-VOC; February 26, 2020 to February 1, 2021), Gamma (February 1, 2021 to June 30, 2021), Delta (June 30, 2021 to November 30, 2021), and Omicron (November 30, 2021 to December 31, 2022).

### Statistical analysis

Descriptive analysis was conducted to compare baseline characteristics between individuals with a single hospitalization and those who were rehospitalized. Categorical variables were expressed as proportions and compared using Pearson’s chi-square test, while continuous variables were assessed for normality using the Kolmogorov–Smirnov test and presented as medians and interquartile ranges (IQR), and compared using the Kruskal–Wallis test. A sensitivity analysis comparing readmissions with and without laboratory-confirmed SARS-CoV-2 infection showed no statistically significant differences; therefore, both case definitions were retained. Missing data were handled by including a separate “missing” category for each variable in the analyses. Rehospitalization rates were calculated annually as the number of rehospitalizations per year divided by the total person-years of follow-up, multiplied by 100.

Survival analysis was performed using the Fine and Gray subdistribution hazards model for competing risks [12], accounting for competing events (such as death) that may preclude the occurrence of the event of interest (rehospitalization). The dominant variant variable was included as a stratification factor to account for temporal variation in circulating variants. This modeling approach allowed for the estimation of subdistribution hazard ratios (sHR) and their respective 95% confidence intervals, adjusted for covariates and time-dependent variables. Model selection was based on the Akaike Information Criterion (AIC). Statistical significance was defined as p < 0.05.

All analyses were conducted using the R environment (version 4.2.3) [13], employing the ***survival*** [14,15] and ***mstate*** [16,17] packages.

### Ethical considerations

This study was approved by the Research Ethics Committee of the National School of Public Health Sergio Arouca, Oswaldo Cruz Foundation, Brazil (ENSP/FIOCRUZ; CAAE: 74953523.5.0000.5240), as well as by the Research Ethics Committee of the Municipal Health Department of Rio de Janeiro (SMS/RJ; CAAE: 74953523.5.3001.5279).

### Patient and public involvement

Patients and the public were not involved in the design, conduct, or reporting of this study.

## Results

The analysis comprised a cohort of 59,001 individuals from the original database, selected according to the established inclusion criteria, of whom 1,957 (3.3%) experienced rehospitalization.

Rehospitalized individuals were more frequently female (52%) and had a higher median age (69 vs. 57 years, p < 0.001), with a greater proportion of older adults (49% aged ≥70 years vs. 33% aged 50–69 years). A higher proportion of black individuals was also observed among rehospitalized patients (45% vs. 37%, p < 0.001), as shown in Table 1.

**Table 1:** Demographic and clinical characteristics of patients with and without rehospitalization in the municipality of Rio de Janeiro, Brazil, 2020–2022.

Characteristics	Total N = 59.001^*1*^	Rehospitalization		
		No N = 57.044^*1*^	Yes N = 1.957^*1*^	p-value^*2*^
**Sex**				**<0.001**
Male	31.309 (53%)	30.361 (53%)	948 (48%)	
Female	27.692 (47%)	26.683 (47%)	1.009 (52%)	
**Age**				**<0.001**
N	59.001	57.044	1.957	
Median (IQR)	58 (45, 70)	57 (44, 70)	69 (56, 80)	
**Age group (years)**				**<0.001**
18-49	19.869 (34%)	15.525 (34%)	344 (18%)	
50-69	23.833 (40%)	23.187 (41%)	646 (33%)	
70 or older	25.269 (26%)	14.302 (25%)	967 (49%)	
**Race/skin color**				**<0.001**
White	12.037 (21%)	11.516 (21%)	521 (27%)	
Black	21.556 (37%)	20.684 (37%)	872 (45%)	
Other	24.101 (42%)	23.540 (42%)	561 (28%)	
**Comorbidities**				**<0.001**
None	22.963 (39%)	22.452 (39%)	511 (26%)	
One	19.398 (33%)	18.830 (33%)	568 (29%)	
Two	11.955 (20%)	11.442 (20%)	513 (26%)	
Three or more	4.685 (8%)	4.320 (8%)	365 (19%)	
**Final classification**				**<0.001**
Unspecified SARI	10.192 (17%)	9.554 (17%)	638 (33%)	
SARS-CoV-2 confirmed SARI	46.486 (79%)	45.215 (79%)	1.271 (65%)	
Missing	2.323 (4%)	2.275 (4%)	48 (2%)	
**ICU admission**				**<0.001**
Yes	23.493 (45%)	22.716 (45%)	777 (49%)	
No	25.623 (49%)	24.926 (49%)	697 (44%)	
Missing	3.096 (6%)	3.000 (6%)	96 (7%)	
**Hospital discharge disposition**				**<0.001**
Cure	41.123 (93%)	40.304 (95%)	819 (51%)	
Death due to COVID-19	1.923 (4.4%)	1.292 (2.7%)	721 (44%)	
Death due to other causes	44 (0.1%)	11 (0.1%)	33 (2%)	
Missing	1.107 (2.5%)	1.059 (2.2%)	48 (3%)	

^*1*^ n (%).

^*2*^ Pearson’s chi-square test; Kruskal-Wallis test.

Rehospitalized patients presented a higher burden of comorbidities, with 19% having three or more pre-existing conditions, compared to 8% among those not rehospitalized. A greater proportion of unspecified severe acute respiratory illness (SARI) was observed in this group (33% vs. 17%, p < 0.001). Mortality (44% vs. 2.7%, p < 0.001) and ICU admission (49% vs. 45%, p < 0.001) were also more frequent among rehospitalized individuals.

Regarding vaccination status, 81% of non-rehospitalized patients had not received any vaccine prior to their first hospitalization, whereas this proportion was lower among rehospitalized patients (49%).

The municipality of Rio de Janeiro reported decreasing COVID-19 rehospitalization rates over time: 5.57 (2020), 2.08 (2021), and 0.72 (2022) per 100 person-years.

Table 2 presents the final model, considering death as a competing event. A complete vaccination regimen including booster doses was associated with a 39% reduction in the risk of rehospitalization compared to unvaccinated individuals and those without booster doses.

**Table 2:** Risk of rehospitalization due to SARS-CoV-2 reinfection, municipality of Rio de Janeiro, 2020–2022.

Variables	sHR^*1*^	CI^*2*^
**Age group (REF = 18–49 years)**		
50-69 years	1.25	1.07-1.45
70 years or older	2.68	2.30-3.12
**Race/color (REF = white)**		
Black	1.51	1.33-1.72
Others	0.72	0.63-0.83
**Comorbidities (REF = none)**		
One	1.46	1.27-1.69
Two	1.85	1.59-2.16
Three or more	3.36	2.84-3.96
**ICU admission (REF = yes)**		
No	0.82	0.74-0.91
Missing	1.11	0.90-1.37
**Vaccination Scheme (REF = Unvaccinated / No booster)**		
Complete scheme with booster	0.61	0.52-0.73

^*1*^Hazard Ratio.

^*2*^Confidence Interval.

The dominant variant variable was used to stratify the model to satisfy the proportional hazards assumption. Although not shown in Table 2, this stratification accounts for temporal variation in circulating variants.

A progressively increasing risk gradient for rehospitalization was observed according to the number of comorbidities: 46% higher for one comorbidity, 85% for two, and 236% for three or more, compared to individuals without comorbidities.

Age was positively associated with the risk of rehospitalization: individuals aged 50–69 and ≥70 years had increased risks of 25% and 168%, respectively, compared to those aged 18–49 years.

Regarding race/skin color, black individuals had a 51% higher risk of rehospitalization, while those classified as “other” had a 28% lower risk, both compared to white individuals. Additionally, not being admitted to the ICU during the first hospitalization was associated with an 18% lower risk of rehospitalization.

## Discussion

This study identified factors associated with SARS-CoV-2 reinfection-related rehospitalization among hospitalized patients in the municipality of Rio de Janeiro, highlighting associations with both biological and sociodemographic factors. A complete vaccination schedule including booster doses was associated with a 39% reduction in the risk of rehospitalization, while advanced age, multimorbidity, and Black race were associated with increased risk. These findings suggest that both biological and sociodemographic factors may contribute to differences in COVID-19 outcomes.

Our findings expand the existing literature by demonstrating that vaccine protection is not limited to primary outcomes such as hospitalization and mortality, but also extends to rehospitalization due to reinfection, an outcome that remains relatively understudied. Previous studies have shown increased protection with additional doses, and our results complement this evidence by highlighting this effect in the context of rehospitalization. This conclusion is supported by national and international evidence: recent Brazilian data show that each additional dose conferred increasing protection against ICU admission, mechanical ventilation, and death, even in elderly individuals with comorbidities [18], while global studies report reductions of 29% and 50% in hospitalization risk with two and three doses, respectively [19,20], with clinical severity predominantly higher among the unvaccinated [21]. Additionally, post-infection protection also proved relevant, reducing hospitalizations, rehospitalizations, and post-acute sequelae [22,23], with booster doses further enhancing defense against reinfections [24–26], reinforcing the findings of the present study.

The occurrence of severe outcomes among vaccinated individuals does not contradict the protective effect observed in this study. Rather, it can be partly attributed to waning immunity over time and the circulation of new SARS-CoV-2 variants [27,28]. Studies show that booster doses restore vaccine effectiveness [29–31], underscoring their importance in preventing severe cases. Still, coverage remains suboptimal: according to data from the EpiRio Panel (accessed in june 2025), only 63.5% of Rio de Janeiro’s population had received a booster dose [32]. This low uptake, combined with vaccine hesitancy [33] and inequalities in access [34], increases the risk of adverse outcomes and facilitates the emergence of new variants and outbreaks.

Advanced age and comorbidities are established risk factors associated with COVID-19 rehospitalization due to reinfection, also demonstrated by Colaneri *et al.* (2025) [35]. A progressive increase in risk is observed from the age of 50 onward, as demonstrated in multiple studies [36–38]. This pattern can be explained by immunosenescence, the physiological decline in immune response associated with aging [39,40], which impairs viral clearance, increases susceptibility to complications [41,42], and accelerates the progression of chronic conditions [43].

It is known that the simultaneous presence of multiple comorbidities contributes to a pro-inflammatory state and elevated risk of cytokine storm [44], while immunosenescence hinders infection control [45]. Our findings corroborate this pattern, revealing a 3.34-fold increased risk of rehospitalization among patients with three or more comorbidities, a result consistent with previous cohort studies [46–48], reinforcing the risk gradient according to the number of pre-existing conditions in relation to rehospitalization.

Concerning race/skin color, black race emerged as a risk factor for reinfection-related rehospitalization, consistent with prior data showing black patients having higher risk of rehospitalization [49,50] and higher mortality and hospitalization rates in Brazil [51,52]. These disparities may reflect the complex intersections between race, socioeconomic status, and healthcare access [53], especially in contexts such as Rio de Janeiro, where 22% of the population live in high social vulnerability areas (favelas) [54]. The higher prevalence of comorbidities in rehospitalized black patients [55] may also contribute to the observed disparities. These findings are consistent with a syndemic perspective on COVID-19, in which social inequities and preexisting vulnerabilities may interact to influence disease outcomes, particularly among socially disadvantaged populations. Although racial disparities have been identified, the unexpected protective effect in the “Others” category should be noted, as it includes many cases with missing race data and a few Indigenous and Asian individuals.

No ICU admission during the initial hospital stay was associated with a lower risk of rehospitalization. One possible explanation is that patients who did not require intensive care had less severe disease during the initial hospitalization. ICU admission is typically for critical cases, while general wards handle less severe ones [56,57]. Additionally, SARS-CoV-2 reinfections are often milder, particularly in individuals fully vaccinated with boosters [58,59].

This study has some limitations. The use of administrative databases may be subject to underreporting and inconsistencies; however, the integration of four complementary data sources enabled cross-validation of the information. The definition of reinfection based on a minimum interval of 90 days does not fully capture the complexity of individual immune responses. In addition, virological clearance between episodes could not be confirmed for all individuals because longitudinal testing data were not consistently available in the databases. Nevertheless, it constitutes a standardized, conservative criterion validated by the Brazilian Ministry of Health, helping to minimize the misclassification of persistent infections as reinfections. Since this definition introduces a bias, immortal time bias, the strategy of initiating follow-up only after this 90-day interval was adopted. This approach was essential to reduce potential biases and enhance the robustness of the results. The ecological classification of viral variants provides an aggregated view of the epidemiological context and may not accurately reflect each patient’s actual exposure to the circulating strain at the time of reinfection. Nevertheless, this approach is widely employed in observational studies, especially in settings where individual-level genomic sequencing data is unavailable. Regarding sample classification, hospitalizations without COVID-19 confirmation (such as unspecified severe acute respiratory syndrome) were included because this classification may have resulted from diagnostic problems and delays or failures in notifications during the pandemic [60]. Finally, the absence of individual-level socioeconomic variables may have resulted in residual socioeconomic confounding and limits a more in-depth analysis of social inequalities. However, this limitation was partially addressed through the inclusion of race/skin color, which is recognized as a valid proxy for inequality in the Brazilian context [61].

Despite these limitations, the study offers important methodological strengths. The retrospective cohort design with a large sample size, combined with linkage of four official databases, provided robust statistical power and population representativeness. Additionally, this linkage improved the completeness and accuracy of the information, enabling more reliable analyses. The statistical analysis employed advanced methods, including Fine & Gray competing risks models [12], which appropriately adjusted for death as competing events. Inclusion of individual vaccination variables obtained from the national immunization system enabled real-world vaccine effectiveness evaluation. Furthermore, focusing on reinfection-related rehospitalization fills a critical gap in the literature, which often addresses primary outcomes or rehospitalizations for other causes, providing evidence to guide booster vaccination policies, especially in higher-risk groups. Future studies should incorporate more detailed socioeconomic variables and assess long-term outcomes, to further advance the understanding of health inequalities and the lasting impacts of COVID-19.

## Conclusion

In summary, this study identified factors associated with SARS-CoV-2 reinfection-related rehospitalization in Rio de Janeiro. A complete vaccination schedule including booster doses was associated with a reduced risk of rehospitalization, while advanced age, multimorbidity, and racial disparities were associated with increased risk. These findings reinforce the role of biological and social determinants in COVID-19 outcomes and highlight the relevance of vaccination status in the context of rehospitalization. Future studies should further explore socioeconomic factors and long-term outcomes to better understand the impacts of the disease, as well as to inform preparedness and response strategies for future respiratory public health emergencies.

## Supporting information

S1 File Supplementary material 1. (DOC)
