# Supplementary material for: Rehospitalization due to COVID-19 reinfection in Rio de Janeiro, 2020–2022: Associations with vaccination status, sociodemographic and clinical factors
Source: PLoS One. 2026 Oct 1;21(10):e0332281. doi: 10.1371/journal.pone.0332281 (PMC13630228; doi:10.1371/journal.pone.0332281)
Supplement: Supplementary file 1 [file pone.0332281.s002.pdf]

Review

Manuscript Number PONE-D-25-45468

"Annual rehospitalization rates due to COVID-19 reinfection in Rio de Janeiro: the role of vaccination and associated factors"

Although the article addresses a very important topic: the rehospitalization of COVID-19 patients, it does not appear to be within the scope of the journal. The analyses are interesting, but they present few results for a PLOS One publication. Another point I consider flawed is the assertion that people of African descent have a higher chance of rehospitalization. As the article itself points out, the results highlight the intricate interplay between biological and social determinants in COVID-19 outcomes. The data do not clarify whether the increased risk of rehospitalization is due to race or poorer socioeconomic conditions.
