# Supplementary material for: Rehospitalization due to COVID-19 reinfection in Rio de Janeiro, 2020–2022: Associations with vaccination status, sociodemographic and clinical factors
Source: PLoS One. 2026 Oct 1;21(10):e0332281. doi: 10.1371/journal.pone.0332281 (PMC13630228; doi:10.1371/journal.pone.0332281)
Supplement: Supplementary file 2 [file pone.0332281.s004.pdf]

Article: Annual rehospitalization rates due to COVID-19 reinfection in Rio de Janeiro (2020–2022): vaccination status and associated factors

Responses to Reviewers

- **Overview and general comments**

We thank the reviewer for the thoughtful and constructive comments and for recognizing the novelty of our study.

Regarding the distinction between reinfection-related rehospitalization and post-COVID condition (long COVID), it is important to emphasize that our study specifically focused on acute SARS-CoV-2 reinfection requiring a new hospitalization, defined using a standardized epidemiological criterion of ≥90 days between symptom onset of the first and subsequent episodes, in accordance with the Brazilian Ministry of Health guidelines. This operational definition is widely used in epidemiological studies to differentiate new infection episodes from prolonged viral shedding or complications related to the initial infection.

In contrast, long COVID (post-COVID condition) refers to a constellation of persistent or new symptoms occurring after the acute phase of infection, without necessarily requiring a new infection event or hospitalization. While emerging evidence suggests potential overlaps in immunological mechanisms, including autoimmune phenomena, these conditions represent distinct clinical and epidemiological entities, with different definitions, timelines, and outcome measures.

Our study design, based on routinely collected administrative data, does not allow for the clinical characterization required to identify long COVID phenotypes. Therefore, our analysis was intentionally restricted to objective, time-defined rehospitalization events, ensuring methodological rigor and reproducibility. We acknowledge that the relationship between reinfection and long COVID is an important and evolving area of research; however, it falls beyond the scope of the present study.

Major comments

I. Title, abstract and keywords

Thank you for this constructive comment. We considered the points raised and revised the manuscript accordingly.

Regarding the emphasis on vaccination, we agree that our results indicate a more pronounced protective effect associated with booster doses compared to primary vaccination alone. However, vaccination status was analyzed considering different levels of exposure (unvaccinated, primary vaccination without booster, and vaccination including booster doses), allowing for an assessment of its impact on rehospitalization due to reinfection.

To improve precision and avoid generalizations, we revised the title to explicitly include the term “vaccination status,” which reflects how this variable was operationalized in our analyses, while maintaining its epidemiological relevance. In addition, we included the 2020–2022 period, improving clarity regarding the temporal scope of the annual rehospitalization rates, and modified the text in lines 1–2 to: *"Annual rehospitalization rates due to COVID-19 reinfection in Rio de Janeiro (2020–2022): vaccination status and associated factors"*.

With respect to the presence of multiple associated factors, we clarify that our intention was not to overemphasize vaccination, but rather to highlight it as a modifiable and policy-relevant factor, particularly in the context of rehospitalization due to reinfection, which remains a relatively understudied outcome.

II. Introduction

Thank you for the suggestion. We revised the Introduction to better emphasize the novelty and originality of the study, ensuring greater alignment with the study objectives and the reviewer’s comments. These revisions were made while maintaining the section within the recommended length, with changes implemented in lines 83–87: *"Monitoring COVID-19 rehospitalizations due to reinfection provides an important opportunity to assess vaccine-conferred protection beyond primary outcomes and to identify more vulnerable populations. This outcome is particularly relevant as it captures both disease severity and healthcare system burden over time"*.

In addition, we incorporated the suggested references to strengthen the contextualization of regional data, including the integration of evidence related to the Brazilian context, in lines 70–81: *"Recent studies indicate that Brazil accounted for a substantial proportion of COVID-19 cases in South America, in a context marked by significant regional inequalities in the availability of healthcare resources[5]. These structural limitations contributed to variations in case severity and clinical outcomes across different regions of the country. In the municipality of Rio de Janeiro, more than*

1.2 million confirmed cases, nearly 20% of the population and 38 thousand deaths were recorded by December 2022, alongside more than 18 million vaccine doses administered[6]. In this context, there is limited evidence integrating linked clinical, epidemiological, and vaccination data to specifically examine rehospitalization due to reinfection, particularly in large urban centers, and it remains insufficiently explored to what extent different vaccination statuses, including booster doses, influence the risk of rehospitalization following reinfection".

III. Materials and Methods

Thank you for these detailed and constructive comments. We have carefully revised the Materials and Methods section to improve clarity, transparency, and alignment with reporting standards.

a) Reporting guidelines (STROBE)

Thank you for this suggestion. We have added a statement at the beginning of the Methods section indicating that the study follows the STROBE guidelines, and we have included the corresponding checklist as Supplementary Material, in lines 98-100: *"This study was reported in accordance with the STROBE (Strengthening the Reporting of Observational Studies in Epidemiology) guidelines, and the corresponding checklist is provided as Supplementary Material."*

b) Study design (explanatory vs predictive)

We have revised the study design description to explicitly state that this is an explanatory cohort study, clarifying the analytical framework and the interpretation of associations, in line 103: *"This is a retrospective explanatory cohort study based on linked administrative databases [...]"*. Additionally, we reviewed the manuscript to avoid causal language, consistently referring to "factors associated with" the outcome.

c) Exposures, outcome, and follow-up definition

We have clarified the analytical structure of the study by explicitly stating that multiple exposures were evaluated, including demographic, clinical, vaccination-related, and contextual variables, in lines 143-145: *"The study evaluated multiple exposures, including demographic, clinical, vaccination-related, and contextual factors, in relation to the risk of rehospitalization due to COVID-19 reinfection."*

We have also redefined the outcome to improve clarity, specifying that it corresponds to time to first rehospitalization due to COVID-19 reinfection, in lines 135-136: *"The primary outcome was time to first rehospitalization due to COVID-19 reinfection."*

In addition, we have defined the start and end of follow-up, stating that follow-up begins 91 days after the first hospitalization and continues until reinfection, death (as a competing event), or administrative censoring, in lines 138-141: *"Follow-up started 91 days after this date and continued until the first occurrence of: (i) symptom onset for rehospitalization due to reinfection, (ii) death (treated as a competing event), or (iii) administrative censoring at the end of the study period (December 31, 2022), whichever occurred first."* These changes were made to eliminate ambiguity regarding the temporal structure of the cohort.

d) Role of vaccination in the analysis

We have clarified that vaccination status was included as one of several exposures, rather than being treated as the sole exposure of interest. This clarification aligns the analytical framework with the multivariable modeling approach used in the study.

e) Follow-up duration clarification

We have clarified that the study does not assume a fixed one-year follow-up, but instead uses a time-to-event framework. The revised text defines the follow-up period and censoring criteria to avoid misinterpretation.

f) Race/skin color classification

We have clarified that race/skin color classification follows the Brazilian Institute of Geography and Statistics (IBGE) standards, and that the "Black" category includes individuals self-identified as Black or Brown. This clarification was added to ensure proper contextualization of race in the Brazilian setting, in lines 147-149: *"race/skin color was classified according to Brazilian Institute of Geography and Statistics (IBGE) categories as White; Black (including individuals self-identified as Black or Brown); and Others (including Yellow, Indigenous, or missing)*."

g) Study population and sample size

We have added a statement clarifying that the study used a population-based approach, including all eligible individuals from the linked databases. Accordingly, we explicitly stated that no a priori sample size calculation was performed, in lines 107-109: *"The study included all eligible individuals identified in the linked databases; therefore, no a priori sample size calculation was performed"*.

h) Statistical analysis transparency (SAMPL)

We have expanded the statistical analysis section to improve transparency, including:

- Normality testing (Kolmogorov–Smirnov test) – line 177: "*Categorical variables were expressed as proportions and compared using Pearson's chi-square test, while continuous variables were tested for normality using the Kolmogorov–Smirnov test and presented as medians and interquartile ranges (IQR) and analyzed using the Kruskal-Wallis test."*
- Explicit definition of effect measures (subdistribution hazard ratios – sHR) – line 186: *"This modeling approach allowed for the estimation of subdistribution hazard ratios (sHR) and their respective 95% confidence intervals [...]"*
- Description of model assumptions (Schoenfeld residuals) – line 188: *"The proportional hazards assumption was assessed using Schoenfeld residuals."*
- Model selection criteria (AIC) – line 189: *"Model selection was based on the Akaike Information Criterion (AIC)."*
- Statistical significance threshold ($p < 0.05$) – lines 189-190: *"Statistical significance was defined as $p < 0.05$."*

i) Ethical considerations

We have added a new subsection explicitly stating that patients and the public were not involved in the study design, conduct, or reporting, in lines 201-203: *"Patients and the public were not involved in the design, conduct, or reporting of this study."*

IV. Results

Thank you for this important suggestion. We have revised the Results section to improve its organization and clarity.

Specifically, the section now begins with a clear description of the participants' baseline characteristics, including demographic and clinical variables, as presented in Table 1. We then present the descriptive findings related to rehospitalization rates over time. Finally, we report the results of the main analysis, including the multiple competing risks model assessing factors associated with rehospitalization due to reinfection (Table 2).

As this study did not include secondary outcomes, no additional analyses are presented in this regard.

These revisions can be found in the Results section (pages 9-12).

VI. Discussion

We thank the reviewer for this suggestion. We revised the Discussion section to ensure better alignment with the Methods and Results, improving the clarity and consistency of the interpretation of findings. We also expanded the description of the study limitations, explicitly addressing potential sources of bias, as well as their direction and magnitude, in accordance with recommendations for cohort studies, as presented in lines 317–337. In addition, we strengthened the section by more explicitly incorporating implications for public health policy and clinical practice, particularly regarding the importance of booster vaccination strategies in reducing severe outcomes such as rehospitalization, as described in lines 346–348. Finally, we included recommendations for future research, highlighting the need to incorporate more detailed socioeconomic variables and to assess long-term outcomes in order to further advance the understanding of health inequalities and the lasting impacts of COVID-19, as stated in lines 349–351: *"Future studies should incorporate more detailed socioeconomic variables and assess long-term outcomes, to further advance the understanding of health inequalities and the lasting impacts of COVID-19."*

V. Conclusion

Thank you for this valuable suggestion. We have revised the Conclusion section to make it more concise and focused on the main findings of the study, as recommended. Specifically, we removed extended policy-oriented statements and relocated these elements to the Discussion section.

The revised Conclusion now emphasizes the key message regarding the association between vaccination status—particularly booster doses—and the risk of rehospitalization due to reinfection, as well as the role of demographic and clinical factors. Additionally, we included a brief perspective on future research, highlighting the need to further investigate socioeconomic factors and long-term outcomes, as well as to inform preparedness for future respiratory public health emergencies, in lines 355-363: *"In summary, this study identified factors associated with SARS-CoV-2 reinfection-related rehospitalization in Rio de Janeiro. A complete vaccination schedule including booster doses was associated with a reduced risk of rehospitalization, while advanced age, multimorbidity, and racial disparities were associated with increased risk. These findings reinforce the role of biological and social determinants in COVID-19 outcomes and highlight the relevance of vaccination status in the context of rehospitalization. Future studies should further explore socioeconomic factors and long-term outcomes to better understand the impacts of the disease, as well as to inform preparedness and response strategies for future respiratory public health emergencies."*

- **Minor comments**

We thank the reviewer for the suggestions. The manuscript has been revised to ensure full compliance with the author guidelines of PLOS ONE. In this process, we standardized the formatting of references both in the text and in the reference list, revised the Abstract to remove subsections as required by the journal, and eliminated numbering from all sections and subsections. Additionally, the manuscript was aligned with the journal's formatting template to ensure consistency throughout.

Regarding reporting guidelines, we acknowledge the importance of the RECORD (Reporting of studies Conducted using Observational Routinely-collected health Data) statement for studies based on routinely collected data. Although the study was originally reported in accordance with the STROBE guidelines, we have now reviewed the manuscript to ensure alignment with RECORD recommendations. The Methods section provides a detailed description of the data sources and the record linkage procedures, including the variables used and the criteria applied. Furthermore, the variables and outcomes are clearly defined, and the analytical procedures, including data handling, are explicitly described. The Discussion section was also strengthened to

include a more comprehensive assessment of the limitations inherent to administrative databases, addressing potential sources of bias.

Finally, as suggested, we have included all required statements at the end of the manuscript, including data availability, conflicts of interest, and acknowledgments.

- **Review Comments to the Author - Major comments**

1. We appreciate this observation. The inconsistency has been corrected throughout the manuscript. All effect estimates now consistently report a 39% reduction in risk, corresponding to an sHR of 0.61 derived from the fully adjusted Fine & Gray model. We have also explicitly labeled these estimates as subdistribution hazard ratios (sHR) to avoid ambiguity.

2. We thank the reviewer for this important observation. We acknowledge that the inclusion of unspecified severe acute respiratory illness (SARI) cases in the outcome may introduce potential misclassification by capturing events not necessarily related to SARS-CoV-2 reinfection. However, this decision was made considering the epidemiological context in Brazil during the pandemic, where diagnostic limitations, reporting delays, and under-ascertainment of laboratory confirmation were common, particularly in earlier periods.

To assess the impact of potential misclassification, we conducted a sensitivity analysis restricting the outcome to rehospitalizations with laboratory-confirmed SARS-CoV-2 infection. This analysis showed no statistically significant differences compared to the main analysis, supporting the robustness of our findings. Based on these results, we retained both case definitions to preserve sensitivity in identifying relevant events, in lines 178-180: "*A sensitivity analysis comparing rehospitalizations with and without laboratory-confirmed SARS-CoV-2 infection showed no statistically significant differences; therefore, both case definitions were retained.*"

3. We thank the reviewer for this important observation. The aim of the study was to assess the association between race/ethnicity, particularly Black race, and the risk of reinfection-related rehospitalization compared with White individuals. We acknowledge that the handling of missing data may introduce bias; however, the proportion of missing data for this variable was low, reducing the likelihood of substantial distortion of the estimates. Nevertheless, we reported the distribution of

missing data in the descriptive tables and expanded the discussion to address potential bias related to this variable. The inclusion of a directed acyclic graph (DAG) was not undertaken; instead, we explicitly described the rationale for covariate adjustment based on theoretical knowledge and prior literature.

4. Absence of ICU admission during the initial hospitalization was associated with a lower risk of rehospitalization. This finding likely reflects differences in baseline clinical severity, as ICU admission is generally reserved for more critical cases, whereas general wards manage less severe conditions. Therefore, ICU admission should be interpreted as a proxy for disease severity rather than a causal factor. Additionally, SARS-CoV-2 reinfections have been reported to present with milder clinical manifestations, particularly among individuals who are fully vaccinated with booster doses.

5. We thank the reviewer for the comment. The calculation of annual rehospitalization rates, including the definition of person-time denominators and the procedures used, is already described in detail in the Methods section, in lines 182-183: *"Rehospitalization rates were calculated annually as the number of rehospitalizations per year divided by the total person-years of follow-up, multiplied by 100."*

6. We revised the text to clarify that we exclusively used the Fine and Gray model for subdistribution hazards. All estimates were labeled as subdistribution hazard ratios (sHR). We removed the reference to the Schoenfeld test and clarified the procedures used to assess the proportional hazards assumption in the context of competing risks.

7. The Methods section details the modeling of vaccination as a time-dependent variable, including updating vaccination status during follow-up, considering effective immunity 14 days after each dose, and using a counting process data structure. We also clarified the strategies used to avoid immortal time bias.

8. We thank the reviewer for this important comment. The time origin was defined based on the date of symptom onset, as this variable presented greater completeness and consistency across the integrated databases. In contrast, hospital

admission and discharge dates showed a high degree of missingness and inconsistencies, which could introduce bias and compromise the reliability of time-to-event estimates.

To ensure internal consistency, both the start of follow-up (91 days after symptom onset) and the definition of the event (time to rehospitalization) were anchored to the same temporal reference (symptom onset). This approach allowed for a standardized and comparable measurement of time at risk across individuals.

We acknowledge that this choice differs from approaches based on hospital admission dates, and this limitation has been explicitly addressed in the Discussion section.

Additionally, this strategy helped minimize potential biases arising from incomplete hospitalization records, ensuring a more robust definition of the risk period.

9. We revised the Methods section to explicitly specify the set of covariates pre-defined based on epidemiological knowledge (age, sex, race/skin color, comorbidities, baseline severity, calendar period/variants, and vaccination). The use of the Akaike Information Criterion (AIC) was retained as a complementary approach and is now described as an exploratory/sensitivity analysis.

10. We thank the reviewer for this important comment. The record linkage process was conducted by the coordinating research team prior to data access, using identifiable information under appropriate ethical and institutional approvals. The dataset made available for this analysis was fully de-identified and did not include linkage keys or individual-level identifiers.

As a result, it was not possible to directly assess linkage performance metrics (such as match rates or false-positive estimates), nor to independently replicate the linkage procedures. Similarly, deduplication of individuals and the definition of hospitalization sequences (index hospitalization and first rehospitalization) were performed beforehand by the coordinating team, following standardized criteria.

11. We acknowledge the potential for residual confounding. We expanded the Discussion section to explicitly address this limitation and included available proxies, such as race/skin color. We also highlight the need for future studies to incorporate

more detailed socioeconomic variables and to apply more robust quantitative methods to better control for confounding.

12. We revised the Data Availability section to include more detailed information, specifying the responsible authority (Epidemiological Intelligence Center – CIE), the access criteria, and the institutional contact, in accordance with the journal’s policy.

13. All references have been reviewed and standardized according to the journal’s style. We corrected inconsistencies in volume, issue, and pagination, and included DOIs whenever available.

14. The manuscript structure was thoroughly revised to comply with the journal’s guidelines, removing inappropriate numbering and standardizing headings, subheadings, and figure captions.

15. We included 95% confidence intervals for the main estimates.

16. We standardized the use of the term “rehospitalization” throughout the manuscript, as well as the nomenclature for race/skin color, ensuring textual consistency.

17. We conducted a comprehensive revision of the manuscript, correcting grammatical inconsistencies, punctuation, hyphenation, and clarity of expressions.

18. The manuscript was fully revised to ensure consistency, clarity, and compliance with the journal’s guidelines, including formal and scientific writing aspects.

We sincerely thank the reviewers for their valuable comments and suggestions, which contributed to improving the clarity and quality of the manuscript. We have carefully addressed all major concerns raised. Minor comments and editorial suggestions have already been incorporated into the revised version of the manuscript.

Documento assinado digitalmente
RANNA KISSIA ALVES DAS NEVES
Data: 30/04/2026 00:08:39-0300
Verifique em https://validar.iti.gov.br

Ranna Kíssia Alves das Neves

Graduate Student, Public Health Epidemiology Program, ENSP/FIOCRUZ

Rio de Janeiro, RJ, Brazil

rannaneves@gmail.com | +55 68 99981-9498
