# Supplementary material for: Rehospitalization due to COVID-19 reinfection in Rio de Janeiro, 2020–2022: Associations with vaccination status, sociodemographic and clinical factors
Source: PLoS One. 2026 Oct 1;21(10):e0332281. doi: 10.1371/journal.pone.0332281 (PMC13630228; doi:10.1371/journal.pone.0332281)
Supplement: Supplementary file 3 [file pone.0332281.s005.pdf]

Article: Rehospitalization due to COVID-19 reinfection in Rio de Janeiro, 2020–2022: associations with vaccination status and sociodemographic and clinical factors

Responses to Reviewers

We thank the reviewer for the thoughtful and constructive comments and for recognizing the novelty of our study.

Comment II:

Thank you for this observation. We agree that the original sentence was grammatically incomplete. We have revised the sentence to clearly state the definition of COVID-19 reinfection adopted in the study. This change has been incorporated into the revised manuscript (Lines 82–84), as follows:

"*According to the technical definition adopted in Brazil, COVID-19 reinfection was defined as a new symptomatic episode occurring at least 90 days after the first infection"*.

Comment III:

Thank you for this important observation. We agree that biological evidence of SARS-CoV-2 clearance between episodes would provide greater certainty in identifying true reinfections. However, in our study, it was not possible to confirm virological clearance for all individuals because longitudinal testing data between episodes were not consistently available in the administrative databases used. Therefore, reinfection was operationally defined using the minimum 90-day interval recommended by the Brazilian Ministry of Health.

In accordance with the reviewer's suggestion, we have acknowledged the absence of confirmation of virological clearance as a limitation in the Discussion section (Lines 323-325). The following sentence was added:

"In addition, virological clearance between episodes could not be confirmed for all individuals because longitudinal testing data were not consistently available in the databases."

Comment IV:

Thank you for this important observation. We carefully revised the Discussion section to avoid statements that could imply causal relationships beyond our findings and

to more clearly distinguish the associations observed in our study from potential explanations supported by previous literature.

The statements were revised as follows:

Lines 258-260: "This study identified factors associated with SARS-CoV-2 reinfection-related rehospitalization among hospitalized patients in the municipality of Rio de Janeiro, highlighting associations with both biological and sociodemographic factors."

Lines 263-264: "These findings suggest that both biological and sociodemographic factors may contribute to differences in COVID-19 outcomes."

Lines 295-297: "It is known that the simultaneous presence of multiple comorbidities contributes to a pro-inflammatory state and elevated risk of cytokine storm[44], while immunosenescence hinders infection control".

Lines 305-311: "These disparities may reflect the complex intersections between race, socioeconomic status, and healthcare access[53], especially in contexts such as Rio de Janeiro, where 22% of the population live in high social vulnerability areas (favelas)[54]. The higher prevalence of comorbidities in rehospitalized black patients[55] may also contribute to the observed disparities. These findings are consistent with a syndemic perspective on COVID-19, in which social inequities and preexisting vulnerabilities may interact to influence disease outcomes, particularly among socially disadvantaged populations."

Lines 315-317: "No ICU admission during the initial hospital stay was associated with a lower risk of rehospitalization. One possible explanation is that patients who did not require intensive care had less severe disease during the initial hospitalization".

Lines 325-327: "In addition, virological clearance between episodes could not be confirmed for all individuals because longitudinal testing data were not consistently available in the databases".

Comment V:

Thank you for this suggestion. We agree that the terms "annual" and "rates" in the previous title did not adequately reflect the primary focus and analytical approach of the study.

Accordingly, we revised the title to better reflect the study design, outcome, and main explanatory variables. We also chose the term "associations" rather than "effect" to avoid implying causality, considering the observational nature of the study.

The revised title (Lines 1–3) is:

"Rehospitalization due to COVID-19 reinfection in Rio de Janeiro, 2020–2022: associations with vaccination status and sociodemographic and clinical factors."

Response to Reviewer #3

We thank the reviewer for the careful and constructive evaluation of our manuscript and for the positive assessment of its methodological approach, statistical analysis, and public health relevance. We carefully considered the comments and suggestions provided and reviewed the manuscript accordingly, with particular attention to methodological clarity, interpretation of the findings, and the limitations of the study.

In particular, we agree that the absence of individual-level socioeconomic information may result in residual confounding and should be more explicitly acknowledged. Therefore, we revised the limitations section of the Discussion (Lines 340-342) as follows:

"Finally, the absence of individual-level socioeconomic variables may have resulted in residual socioeconomic confounding and limits a more in-depth analysis of social inequalities."

We believe that this revision, together with the other language and interpretative refinements made throughout the manuscript, provides a more cautious interpretation of the findings and improves the overall clarity of the Discussion.

Documento assinado digitalmente
RANNA KISSIA ALVES DAS NEVES
Data: 14/08/2026 22:45:53-0300
Verifique em https://validar.iti.gov.br

Ranna Kíssia Alves das Neves

Graduate Student, Public Health Epidemiology Program, ENSP/FIOCRUZ

Rio de Janeiro, RJ, Brazil

rannaneves@gmail.com | +55 68 99981-9498
