# Supplementary material for: Rehospitalization due to COVID-19 reinfection in Rio de Janeiro, 2020–2022: Associations with vaccination status, sociodemographic and clinical factors
Source: PLoS One. 2026 Oct 1;21(10):e0332281. doi: 10.1371/journal.pone.0332281 (PMC13630228; doi:10.1371/journal.pone.0332281)
Supplement: Peer review history

# Peer review: Decision Letter 0

**DOI:** 10.1371/journal.pone.0332281.r001

**Authors:** Mickael Essouma

2 Oct 2025

Dear Dr. Neves,

Thank you for submitting your manuscript to PLOS ONE. After careful consideration, we feel that it has merit but does not fully meet PLOS ONE’s publication criteria as it currently stands. Therefore, we invite you to submit a revised version of the manuscript that addresses the points raised during the review process.

Please submit your revised manuscript by Nov 16 2025 11:59PM. If you will need more time than this to complete your revisions, please reply to this message or contact the journal office at plosone@plos.org. When you're ready to submit your revision, log on to https://www.editorialmanager.com/pone/ and select the 'Submissions Needing Revision' folder to locate your manuscript file.

- A rebuttal letter that responds to each point raised by the academic editor and reviewer(s). You should upload this letter as a separate file labeled 'Response to Reviewers'.
- A marked-up copy of your manuscript that highlights changes made to the original version. You should upload this as a separate file labeled 'Revised Manuscript with Track Changes'.
- An unmarked version of your revised paper without tracked changes. You should upload this as a separate file labeled 'Manuscript'.

If you would like to make changes to your financial disclosure, please include your updated statement in your cover letter. Guidelines for resubmitting your figure files are available below the reviewer comments at the end of this letter.

If applicable, we recommend that you deposit your laboratory protocols in protocols.io to enhance the reproducibility of your results. Protocols.io assigns your protocol its own identifier (DOI) so that it can be cited independently in the future. For instructions see: https://journals.plos.org/plosone/s/submission-guidelines#loc-laboratory-protocols. Additionally, PLOS ONE offers an option for publishing peer-reviewed Lab Protocol articles, which describe protocols hosted on protocols.io. Read more information on sharing protocols at https://plos.org/protocols?utm_medium=editorial-email&utm_source=authorletters&utm_campaign=protocols.

We look forward to receiving your revised manuscript.

Kind regards,

Mickael Essouma, M. D.

Academic Editor

PLOS ONE

Journal Requirements:

When submitting your revision, we need you to address these additional requirements.

1. Please ensure that your manuscript meets PLOS ONE's style requirements, including those for file naming. The PLOS ONE style templates can be found at https://journals.plos.org/plosone/s/file?id=wjVg/PLOSOne_formatting_sample_main_body.pdf and https://journals.plos.org/plosone/s/file?id=ba62/PLOSOne_formatting_sample_title_authors_affiliations.pdf

2. For studies involving third-party data, we encourage authors to share any data specific to their analyses that they can legally distribute. PLOS recognizes, however, that authors may be using third-party data they do not have the rights to share. When third-party data cannot be publicly shared, authors must provide all information necessary for interested researchers to apply to gain access to the data. (https://journals.plos.org/plosone/s/data-availability#loc-acceptable-data-access-restrictions)

For any third-party data that the authors cannot legally distribute, they should include the following information in their Data Availability Statement upon submission:

1) A description of the data set and the third-party source

2) If applicable, verification of permission to use the data set

3) Confirmation of whether the authors received any special privileges in accessing the data that other researchers would not have

4) All necessary contact information others would need to apply to gain access to the data

3. If the reviewer comments include a recommendation to cite specific previously published works, please review and evaluate these publications to determine whether they are relevant and should be cited. There is no requirement to cite these works unless the editor has indicated otherwise.

Additional Editor Comments :

1. Overview and general comments

I commend the authors for conducting a study focusing on first rehospitalization for first acute COVID-19 relapse in Rio de Janeiro, Brazil. The study is innovative and timely because as clearly stated by the authors, relapse of acute COVID-19 is rarely addressed in the literature, unlike first acute COVID-19 and so-called post-COVID condition/long COVID. In the hindsight, it is unclear whether the so-called long COVID/post-COVID is chronic COVID-19 that may start as a relapse of acute COVID-19 (https://www.thelancet.com/action/showPdf?pii=S0140-6736%2824%2901136-X, https://doi.org/10.1080/13814788.2025.2535618), especially since acute COVID-19 and so-called long COVID reportedly present with latent or clinical overt autoimmune phenotypes (https://doi.org/10.1371/journal.pone.0304990, https://doi.org/10.1186/s12879-025-11536-8). However, these bits of information seem to not have been considered when designing this study, and the current version of the manuscript also presents other important methodological and editing issues as highlighted by the reviewers. Below, I specify some methodological and editing aspects that need to be addressed in addition to those highlighted by the reviewers.

2. Major comments

-Title, abstract and keywords. It seems that it is vaccination booster doses (first booster? Second booster?) that were found to be protective, not just vaccination. This should be reflected in the title if the authors wish to emphasize on vaccination in the title. Why emphasize on vaccination when there were so many other factors (positively and negatively) linked with rehospitalization and when COVID-19 efficacy is so much debated in the literature? See Dhayfule et al. BMC Infectious Diseases (2025) 25:1169 https://doi.org/10.1186/s12879-025-11289-4, Monash Bioeth Re. 2024 Jun;42(1):28-54. doi: 10.1007/s40592-024-00189-z). Would it be easy for readers to understand what you mean by «Annual rehospitaliation » if they just read your manuscript's title without going through the full-text? After revising the manuscript's title and the main manuscript, consider integrating those revisions with these guidelines to also revise the abstract and keywords: https://doi.org/10.1098/rspb.2024.1222.

-Introduction. Consider strengthening it to reflect its novelty/originality based on reviewers' comments, the study's aim and as commented above, ideally without exceeding 1.5 page. When presenting regional data, these articles could be useful: https://doi.org/10.1371/journal.pone.0332883, https://doi.org/10.1371/journal.pgph.0003023.

-Materials and Methods. Consider beginning this section with a statement about the reporting guidelines used and uploading the corresponding checklist in the supplemental Material to demonstrate your compliance with those guidelines. Could you complete the sentence in line 92 specifying whether the cohort was predictive or explanatory? The relevance of this specification stems from the fact that it helps assess whether you can or cannot use a «CAUSAL LANGUAGE » in this manuscript. It also helps determine whether the statistical model used for the main analysis was explanatory of predictive, which could ultimately help determine whether or not it is appropriate to name the factors associated with rehospitalization as risk factors. Further guidance on this matter can be found here: https://doi.org/10.1186/s12982-018-0080-z. You claim that the study was a cohort study, but it is unclear going through this section and the Results section what were the exposure, the exposed group, the non-exposed group, the outcomes, and timelines for monitoring the outcome. Notably, would the exposure be a booster dose of vaccination after a first episode of acute COVID-19? If so, would this be the reason why you included the word vaccination in the manuscript's title? Then how did you find that other reportedly associated factors were risk factors when a risk factor is a factor considered to be an exposure in a cohort study (https://www.mdpi.com/2077-0383/13/14/4005, CHEST 2020; 158(1S):S72-S78)? Did you wish to say that the primary outcome was rehospitalization within one year after hospitalization for a first episode of acute COVID-19 in lines 121 and122? If there was a primary outcome, what was/were the secondary outcome(s)? I believe these cohort studies together with the reporting guidelines provided below may help you improve this section as should be seen in a cohort study report: https://doi.org/10.1371/journal.pone.0317686, https://doi.org/10.1371/journal.pone.0330110. You provided a race classification but I do not see a clear ethnic/ancestral classification meanwhile the latter classification is more important in health research. Along this line, I was interested by the fact that so-called Black people were more likely to be rehospitalized (within one year?) after hospitalisation for a first episode of acute COVID-19 due to acute COVID-19 relapse, given the reportedly high burden of acute COVID-19 in African Americans, although long COVID/post-COVID condition is reportedly more diagnosed in White people than in African Americans in US which is in the same continent as Brazil. The authors need to specify whether the Black people highlighted in this study were African Latino Americans as would be expected. Furthermore, did you stratify data by ethnicity and race rather than adjusting the race variable as is recommended (https://doi.org/10.1093/epirev/mxad0100? How did you sample the study participants and what method di you use to estimate the sample size (Oral Oncology Reports 12 (2024) 100662)? When revising the Statistical analysis sub-section as recommended by the reviewers, consider adapting the SAMPL guidelines (https://onlinelibrary.wiley.com/doi/10.1002/9781118715598.ch25) to an appropriate presentation of statistical analyses in a cohort study (https://www.mdpi.com/2077-0383/13/14/4005, CHEST 2020; 158(1S):S72-S78). This means you should also provide information about how data is presented and visualized in the manuscript, as well as details about how effect magnitude estimates were derived, how their 95% confidence intervals were estimated, how statistical significance was defined, and what statistical tests were used to determine whether variables are normally distributed or not. Consider adding information about whether you complied with the Helsinki Declaration in the Ethical approval sub-section whose name should be changed to something like «Ethical considerations». Just after the «Ethical considerations» sub-section, consider adding one sub-section termed «Patient and Public involvement».

-Results. After revising the Materials and Methods section, consider revising it to present first participants' characteristics, then results of the primary outcome, and finally results of secondary outcomes if any.

-Discussion. Consider revising it to match the content of the two sections mentioned just above. Limitations should be recorded keeping in mind standards of cohort studies: https://www.mdpi.com/2077-0383/13/14/4005, CHEST 2020; 158(1S):S72-S78. Did you provide recommendations for policy, clinical practice and future research in this section?

-Conclusion. It is very long and its length would better be reduced to the main take-home message and perspective for future research in this domain, with recommendations moved to the Discussion section.

3. Minor comments

-Manuscript formatting. Conform to PLOS One author guidelines. This is true for reference citing in the text and at the end of the manuscript, the abstract which is not expected to have sub-sections, and section and sub-sections that are not expected to be numbered. The link to access a PLOS One manuscript template is provided above. In addition, it seems that data analyzed were based on routinely collected health data, but it is unclear whether you complied with the corresponding appropriate reporting (RECORD) guidelines: see https://journals.plos.org/plosmedicine/article?id=10.1371/journal.pmed.1001885. Moreover, also add further statements (e.g., conflicts of interest, data availability) at the end of the manuscript in addition to the Acknowledgment statement.

-Language editing is also advised.

Mickael Essouma, M.D.

[Note: HTML markup is below. Please do not edit.]

Reviewers' comments:

Reviewer's Responses to Questions

**Comments to the Author**

1. Is the manuscript technically sound, and do the data support the conclusions?

Reviewer #1: Partly

Reviewer #2: No

Reviewer #3: Yes

Reviewer #4: Partly

**********

2. Has the statistical analysis been performed appropriately and rigorously?

Reviewer #1: I Don't Know

Reviewer #2: No

Reviewer #3: Yes

Reviewer #4: No

**********

3. Have the authors made all data underlying the findings in their manuscript fully available?

The PLOS Data policy

Reviewer #1: No

Reviewer #2: No

Reviewer #3: Yes

Reviewer #4: Yes

**********

4. Is the manuscript presented in an intelligible fashion and written in standard English?

Reviewer #1: Yes

Reviewer #2: No

Reviewer #3: Yes

Reviewer #4: Yes

**********

Reviewer #1: Although the article addresses a very important topic: the rehospitalization of COVID-19 patients, it does not appear to be within the scope of the journal. The analyses are interesting, but they present few results for a PLOS One publication. Another point I consider flawed, is the assertion that people of African descent have a higher chance of rehospitalization. As the article itself points out, the results highlight the intricate interplay between biological and social determinants in COVID-19 outcomes. The data do not clarify whether the increased risk of rehospitalization is due to race or poorer socioeconomic conditions.

Reviewer #2: This retrospective cohort study links four Rio de Janeiro administrative datasets (SIPNI, e-SUS Notifica, SIVEP-Gripe, SIM) to estimate annual rehospitalization rates due to presumed SARS-CoV-2 reinfection (≥90 days after the index episode) in 2020–2022, and to evaluate factors—especially vaccination—associated with time to first rehospitalization, accounting for death as a competing risk. Among 59,001 hospitalized adults, 1,957 (3.3%) were rehospitalized; boosted vaccination (≥3 doses, or ≥2 for Janssen) is reported to reduce risk (HR 0.61, 95% CI 0.52–0.73), whereas older age, multimorbidity, and Black race/skin color increased risk. While timely and relevant, the manuscript has important scientific, methodological, reporting, and formatting issues that currently limit the robustness and reproducibility of the findings.

Major comments

1. Effect size inconsistency (39% vs 36% reduction). The Abstract cites a 39% risk reduction with booster (HR 0.61), but the Discussion states 36%—these must match and derive from the same model (ideally the fully adjusted primary analysis). Align the numbers throughout and state clearly whether these are subdistribution hazard ratios. Standardize to one figure (e.g., “39%”) consistent with HR 0.61, and explicitly label as sHR if using Fine–Gray.

2. Outcome definition may conflate reinfection with unrelated/unspecified SARI. The endpoint allows unspecified SARI at rehospitalization; 33% of rehospitalized cases are “unspecified SARI,” raising misclassification concerns of non-COVID etiologies as “reinfections.” Provide a sensitivity analysis restricted to lab-confirmed COVID-19 at rehospitalization.

3. Race/skin-color handling is problematic. Missing race is grouped into “Others,” potentially biasing associations and the observed “protective” effect in “Others.” Treat missing as a separate category; consider multiple imputation; present distributions of missingness and a directed acyclic graph (DAG) justifying adjustment sets.

4. Interpretation of ICU covariate. The manuscript interprets “no ICU at index stay” as protective; however, ICU reflects baseline severity and service availability, not a causal factor. Frame this as adjustment for severity and avoid causal language.

5. Annual rates lack denominators/uncertainty. The reported per-year rates (2020: 5.57; 2021: 2.08; 2022: 0.72 per 100 person-years) do not show person-time contributions or 95% CIs, and methods for attributing person-time to calendar years are not described. Provide person-years per year, CIs, and exact censoring dates.

6. Competing-risk modeling is ambiguously specified. The text mixes “Cox proportional hazards” with “Fine & Gray,” and mentions testing proportional hazards with Schoenfeld residuals (appropriate for cause-specific Cox, not subdistribution hazards). Clarify whether you estimated cause-specific hazards or subdistribution hazards, label estimates accordingly (HR vs sHR), and use appropriate diagnostics (e.g., time-interaction terms for sHR).

7. Time-varying vaccination not fully specified. You state vaccination is effective ≥14 days post-dose and mention “time-dependent variables,” but do not specify if/when vaccination status was updated during follow-up (after day 91) or how immortal-time was avoided with respect to vaccination exposure. Provide full details (split follow-up intervals, counting process, exposure lags) and a directed timeline schematic.

8. Start of follow-up and event times. You begin follow-up at day 91 after symptom onset to mitigate immortal-time bias; however, the event time is “symptom onset for rehospitalization,” not admission. Confirm consistency (symptom-based origin and event) and report median follow-up, number at risk over time, and cumulative incidence functions (CIF) by vaccination status.

9. Model building and covariate selection. You cite AIC-based selection but do not list candidate models or pre-specified covariates. For transparency, pre-specify the adjustment set (age, sex, race, comorbidity count, index severity, calendar time/variant, etc.), justify inclusion, and move any exploratory selection to sensitivity analyses.

10. Record linkage & data quality. Provide linkage performance (match rates, clerical review, false-link estimates), deduplication of individuals across sources, and how multiple hospitalizations were collapsed to “first” and “first re-.” A short RECORD-linkage appendix or flowchart would help.

11. Confounding by indication and access. Vaccination is associated with socio-demographics and healthcare access; consider additional adjustment (e.g., neighborhood deprivation proxy), propensity weighting, or negative-control analyses if possible, and discuss residual confounding more explicitly.

12. Data Availability does not meet PLOS ONE policy. “Contact via the institution’s official email” is too vague. Provide the exact Data Access Committee name, contact info, and a path for qualified researchers; share a minimally de-identified dataset and code where legally permissible, or give a concrete legal basis for restriction plus a public metadata/code repository.

13. Reference accuracy/formatting. Several entries appear malformed or need DOIs/URLs; check items such as “Vaccines (Basel) 2024;26(12):464” and “Vaccines (Basel). 2022;10(11):1885.21” for correct volume/pages; ensure consistent style, add DOIs where available, and verify all Portuguese titles/availability statements.

14. Numbering and section labels. Inside “2. Materials and Methods,” a subsection is labeled “1.1 Outcome and Variables,” and the figure anchor/file name appears as “Figura … Figure1_paper2 (2).docx.” Standardize English section numbering, and ensure figures are cited and captioned per journal style.

15. Quantities and uncertainty. Add 95% CIs for annual rates, provide a CONSORT-style cohort flow (already referenced), and add CIF plots by vaccination status and comorbidity strata.

16. Terminology consistency. Use either “rehospitalization” or “readmission” consistently (title, Abstract, text, tables). Standardize capitalization of race/ethnicity terms (e.g., “Black,” “White”) per style.

17. Grammar/phrasing clean-up. Examples:

+ “symptoms ≥90 days after the one in the first hospitalization” → “symptom onset ≥90 days after the index episode.”

+ “The main finding was that a complete vaccination regimen with a booster (≥3 doses) was associated with a 39% reduction …” (fine) but avoid later “36%.”

18. Do a careful copy-edit for subject–verb agreement, hyphenation (e.g., “time-dependent”), and punctuation.

Minor comments

19. Define precisely the administrative censoring date and maximum follow-up window; report median [IQR] follow-up and number of events after each dose category.

20. Clarify whether death during the 90-day “washout” period was excluded from the risk set (you imply it, but please state explicitly in figure and text).

21. Consider reporting both cause-specific HRs and subdistribution sHRs for transparency (main text can keep sHRs; cause-specific in supplement).

22. Replace informal labels like “pre-VOC” with a precise date range at first mention; keep all ranges in the same format.

23. Where you discuss post-infection protection, make clear whether those exposures were measured in your data or cited from literature only.

Reviewer #3: This retrospective observational study investigates the annual rate of rehospitalizations due to COVID-19 reinfection in Rio de Janeiro and evaluates the role of vaccination and other associated risk factors.

The study addresses a pressing public health concern, especially in LMICs where both reinfection risk and healthcare system burden remain high. The large sample size, extended time frame, and inclusion of both clinical and vaccination variables are commendable.

That said, the manuscript would benefit from improved methodological clarity, more detailed reporting of statistical analyses, and expanded discussion on the interpretation and implications of its findings.

1. Abstract

Strengths:

Clearly defines the objective and methods.

Reports the rehospitalization rate and main associations.

Recommendations:

Specify study period (e.g., July 2021–July 2022).

Include effect sizes with confidence intervals (e.g., ORs for risk factors).

Clarify what is meant by “reinfection” — laboratory confirmed? Time between episodes?

2. Introduction

Strengths:

Sets up the importance of monitoring reinfection-related hospital burden.

Highlights vaccination as a possible protective factor.

Recommendations:

Include a brief literature review of reinfection rates and outcomes globally and regionally.

Clarify what is novel in this study: Is it the focus on hospitalization? Use of state data? Post-vaccination period?

Define terms like “reinfection” (is it >90 days after the first positive test per CDC definition?).

3. Methods

Strengths:

Large retrospective dataset from a real-world hospital system.

Inclusion of key demographic and clinical variables.

Recommendations:

Reinfection Definition:

Clearly state how reinfections were identified and confirmed: PCR-based? ICD-10 coding?

Was there a minimum time gap between first infection and reinfection?

Population & Sampling:

Report inclusion and exclusion criteria.

Describe whether the cohort includes all patients admitted or only those with lab-confirmed reinfection.

Vaccination Data:

Specify vaccine types, doses, and timing relative to reinfection.

Define what qualifies as “complete vaccination” (2 doses? Boosters?).

Statistical Analysis:

Clearly describe regression models used (e.g., logistic regression).

Report variables adjusted for.

Explain how multicollinearity or missing data were handled.

Ethics:

Mention ethical approval, data anonymization, and waiver of informed consent (if applicable).

4. Results

Strengths:

Reports annual rehospitalization rate (3.4%) and main associated factors.

Includes analysis by comorbidities and vaccination status.

Recommendations:

Tables:

Improve table labels and structure. For example, Table 1 should include denominators and percentages.

Include confidence intervals and p-values alongside odds ratios.

Stratified Results:

Consider presenting stratified results (e.g., by age group, vaccination status, comorbidity burden).

Clarify whether time to reinfection varied by vaccination status.

Effect Size Interpretation:

Help readers interpret findings: e.g., “vaccinated individuals had a 30% lower odds of rehospitalization due to reinfection.”

5. Discussion

Strengths:

Acknowledges protective effect of vaccination and highlights burden on healthcare systems.

Recommendations:

Interpretation:

Expand interpretation of findings in light of prior studies — how do rates in this cohort compare to national or international data?

Mechanisms:

Discuss potential biological or behavioral mechanisms: Why might vaccination reduce rehospitalization — via immune protection, reduction in severe disease, or both?

Policy Implications:

Provide actionable suggestions: e.g., prioritization of booster campaigns, high-risk patient monitoring.

Limitations:

Discuss the lack of genomic confirmation for reinfection.

Acknowledge possible misclassification of infection episodes (e.g., reactivation vs reinfection).

Note absence of some variables (e.g., time from vaccination, immune status, socioeconomic data).

6. Conclusion

Summarizes key findings appropriately.

Suggest specifying policy relevance more directly — e.g., “Our findings support the role of complete vaccination in reducing the burden of COVID-19-related hospital care in reinfection scenarios.”

Reviewer #4: This is a strong, important observational study addressing an under-studied outcome (rehospitalization due to SARS-CoV-2 reinfection) in a large urban population. This retrospective cohort (n=59,001) links four administrative databases in Rio de Janeiro (2020–2022) to estimate annual rehospitalization rates for SARS-CoV-2 reinfection, and to evaluate factors associated with the first rehospitalization ≥90 days after index hospitalization. Using Fine & Gray competing-risk Cox models (death as competing event), the authors report 1,957 rehospitalizations (3.3%). A complete vaccination schedule with booster (≥3 doses, with Janssen handled specially) was associated with a lower hazard of rehospitalization (HR 0.61, 95% CI 0.52–0.73). Older age, multimorbidity and Black race were associated with increased hazard.

Following are my comments:

1. Major comments

A. Causal language & interpretation of vaccine effect

The manuscript sometimes implies causality (e.g., “offered protection” and “reduced risk”). This is an observational study and residual confounding (including healthy-vaccinee bias) is likely. Rephrase to emphasize an association between booster receipt and lower hazard, and discuss key sources of confounding more explicitly.

Strongly consider additional analyses to reduce confounding.

B. Treatment of vaccination as exposure - time-varying nature and immortal time concerns

You state vaccination is considered effective 14 days after last dose, but it is unclear whether vaccination status was modeled as time-varying after cohort entry (91 days after index) or only as a baseline covariate. If vaccination could occur during follow-up, not treating it as time-varying introduces immortal-time bias and misclassification.

Recommendation: treat vaccination (and booster) as a time-dependent covariate with the 14-day lag in the survival model (or, at a minimum, present sensitivity analyses showing results when vaccination is modeled time-updating vs fixed). If time-updated modeling is infeasible, clearly justify and quantify the likely direction/magnitude of bias.

C. Record linkage details and validation

The linkage algorithm is only briefly described. For reproducibility and assessment of bias you should report: deterministic/probabilistic approach, matching variables used, matching thresholds, percent of records matched, clerical review procedures, estimated false-match and missed-match rates, and any validation versus gold-standard samples. If possible, add linkage accuracy metrics in a supplement. Linkage errors could bias both exposure and outcome ascertainment.

D. Definition / ascertainment of reinfection and outcome timing

The 90-day rule is reasonable, but key clarifications are needed: what is the administrative censoring date? Do you include hospitalizations up to 31 Dec 2022 and censor thereafter? How were symptom onset dates handled when missing or inconsistent? How are rehospitalizations due to post-COVID complications (not new infection) distinguished from reinfection?

Recommendation: present sensitivity analyses using alternate reinfection windows (e.g., ≥60 days and ≥120 days) and a restricted analysis including only laboratory-confirmed cases (PCR/antigen) to check robustness.

E. Handling of missing data

Race is collapsed into White / Black (Black + Brown) / Others (including Yellow, Indigenous, missing). Combining missing into “Others” is problematic and can bias estimates. Similarly, other variables may have missing categories (ICU admission = “missing”).

Recommendation: provide a full table of missingness by variable; perform multiple imputation (or at least a complete-case sensitivity analysis). Reclassify race so that missing is a separate category and consider presenting analyses excluding records with missing race as sensitivity.

F. Confounding control and model specification

You used AIC for model selection and stratified by dominant variant to address proportional hazards. Please report which covariates were considered, the final model covariates, and how time-dependent covariates were handled.

Recommendation: in addition to the adjusted Fine & Gray model, consider: (a) propensity score methods or inverse-probability weighting (IPTW) to address measured confounding for vaccination (b) marginal structural models if vaccination is time-varying and affected by time-varying confounding. Also present both subdistribution HRs (Fine & Gray) and cause-specific hazards to aid interpretation.

G. Variant classification and calendar time

The ecological variant periods are reasonable, but individual exposure to variant is uncertain. Calendar time is a powerful confounder (changes in testing, admission thresholds, and vaccination coverage). Stratifying by variant helps, but you should also adjust for calendar time more flexibly (e.g., by week/month splines) or present stratified analyses across variant periods (interaction tests: vaccine effect × variant period).

H. Results presentation: absolute risks and person-time

You report rehospitalization rates per 100 person-years but do not present person-years or 95% CIs for these rates. Provide numerator, denominator (person-years), and CIs. Also present cumulative incidence (%) at fixed timepoints (e.g., at 1 year) by vaccination status - absolute differences improve interpretability.

I. Inconsistencies and numeric errors

There is an inconsistency: Results say “39% reduction (HR 0.61...)” while Discussion says “36% reduction.” Please check and harmonize effect sizes and percent reductions throughout the text.

J. Death frequency & timing needs clarification

You write “Death was more frequent in this group (44% vs. 2.7%, p<0.001).” Is that death during the first hospitalization, death during follow-up, or death at any time? Because death is treated as a competing event, clarify the timing and which deaths are included in these percentages (index hospitalization deaths prior to being eligible for reinfection were presumably excluded). Clarify in Results and Table footnotes.

2. Minor comments / stylistic / reporting suggestions

A. Terminology consistency: use either “rehospitalization” or “readmission” consistently throughout the manuscript. I suggest “rehospitalization due to reinfection” for clarity.

B. Race categories: in Portuguese/IBGE classification, “Amarelo” is usually rendered as “Asian”. Replace “Yellow” with “Asian” if that reflects the IBGE term; explain grouping rationale (why Black + Brown grouped as “Black”).

C. ICU result interpretation: sentence “not being admitted to the ICU … was associated with an 18% lower risk of readmission” reads odd when ICU admission would normally indicate severe index disease. State clearly whether ICU = reference and interpret directionally.

D. Abstract tightening: shorten Methods to 2–3 sentences; report absolute numbers (n events / person-years) and HR with CI for main exposure; avoid over-claiming.

E. STROBE checklist: include a completed STROBE checklist and make sure cohort selection, missing data, linkage, and bias mitigation steps are reported per STROBE.

F. Model diagnostics: report Schoenfeld residual plots / tests for proportional hazards (you say you checked this - show results/plots in supplement). Report discrimination (e.g., c-index) for models if applicable.

G. Vaccine brands & dosing: if available, show stratified analyses by vaccine platform (CoronaVac / AstraZeneca / Pfizer / Janssen) and by time since booster to evaluate waning. If numbers are small, report descriptively and mention as limitation.

H. Grammar / typos: a number of small grammatical issues and punctuation/spacing errors are present (e.g., “69 vs, 57 years, p < 0.001” — remove extra comma/space). Carefully copy-edit the final manuscript.

**********

what does this mean?). If published, this will include your full peer review and any attached files.

If you choose “no”, your identity will remain anonymous but your review may still be made public.

**Do you want your identity to be public for this peer review?**  For information about this choice, including consent withdrawal, please see our Privacy Policy

Reviewer #1: No

Reviewer #2: No

Reviewer #3: **Yes:**  Juan Carlos Alvarado Gonzalez MD

Reviewer #4: No

**********

[NOTE: If reviewer comments were submitted as an attachment file, they will be attached to this email and accessible via the submission site. Please log into your account, locate the manuscript record, and check for the action link "View Attachments". If this link does not appear, there are no attachment files.]

While revising your submission, please upload your figure files to the Preflight Analysis and Conversion Engine (PACE) digital diagnostic tool, https://pacev2.apexcovantage.com/. PACE helps ensure that figures meet PLOS requirements. To use PACE, you must first register as a user. Registration is free. Then, login and navigate to the UPLOAD tab, where you will find detailed instructions on how to use the tool. If you encounter any issues or have any questions when using PACE, please email PLOS at figures@plos.org. Please note that Supporting Information files do not need this step.

Attachment Submitted filename: Review.pdf

# Author comment: Author response to Decision Letter 1

**DOI:** 10.1371/journal.pone.0332281.r002

18 May 2026

Responses to Reviewers

• Overview and general comments

We thank the reviewer for the thoughtful and constructive comments and for recognizing the novelty of our study.

Regarding the distinction between reinfection-related rehospitalization and post-COVID condition (long COVID), it is important to emphasize that our study specifically focused on acute SARS-CoV-2 reinfection requiring a new hospitalization, defined using a standardized epidemiological criterion of ≥90 days between symptom onset of the first and subsequent episodes, in accordance with the Brazilian Ministry of Health guidelines. This operational definition is widely used in epidemiological studies to differentiate new infection episodes from prolonged viral shedding or complications related to the initial infection.

In contrast, long COVID (post-COVID condition) refers to a constellation of persistent or new symptoms occurring after the acute phase of infection, without necessarily requiring a new infection event or hospitalization. While emerging evidence suggests potential overlaps in immunological mechanisms, including autoimmune phenomena, these conditions represent distinct clinical and epidemiological entities, with different definitions, timelines, and outcome measures.

Our study design, based on routinely collected administrative data, does not allow for the clinical characterization required to identify long COVID phenotypes. Therefore, our analysis was intentionally restricted to objective, time-defined rehospitalization events, ensuring methodological rigor and reproducibility. We acknowledge that the relationship between reinfection and long COVID is an important and evolving area of research; however, it falls beyond the scope of the present study.

Major comments

I. Title, abstract and keywords

Thank you for this constructive comment. We considered the points raised and revised the manuscript accordingly.

Regarding the emphasis on vaccination, we agree that our results indicate a more pronounced protective effect associated with booster doses compared to primary vaccination alone. However, vaccination status was analyzed considering different levels of exposure (unvaccinated, primary vaccination without booster, and vaccination including booster doses), allowing for an assessment of its impact on rehospitalization due to reinfection.

To improve precision and avoid generalizations, we revised the title to explicitly include the term “vaccination status,” which reflects how this variable was operationalized in our analyses, while maintaining its epidemiological relevance. In addition, we included the 2020–2022 period, improving clarity regarding the temporal scope of the annual rehospitalization rates, and modified the text in lines 1–2 to: "Annual rehospitalization rates due to COVID-19 reinfection in Rio de Janeiro (2020–2022): vaccination status and associated factors".

With respect to the presence of multiple associated factors, we clarify that our intention was not to overemphasize vaccination, but rather to highlight it as a modifiable and policy-relevant factor, particularly in the context of rehospitalization due to reinfection, which remains a relatively understudied outcome.

II. Introduction

Thank you for the suggestion. We revised the Introduction to better emphasize the novelty and originality of the study, ensuring greater alignment with the study objectives and the reviewer’s comments. These revisions were made while maintaining the section within the recommended length, with changes implemented in lines 83–87: "Monitoring COVID-19 rehospitalizations due to reinfection provides an important opportunity to assess vaccine-conferred protection beyond primary outcomes and to identify more vulnerable populations. This outcome is particularly relevant as it captures both disease severity and healthcare system burden over time".

In addition, we incorporated the suggested references to strengthen the contextualization of regional data, including the integration of evidence related to the Brazilian context, in lines 70–81: "Recent studies indicate that Brazil accounted for a substantial proportion of COVID-19 cases in South America, in a context marked by significant regional inequalities in the availability of healthcare resources[5]. These structural limitations contributed to variations in case severity and clinical outcomes across different regions of the country. In the municipality of Rio de Janeiro, more than 1.2 million confirmed cases, nearly 20% of the population and 38 thousand deaths were recorded by December 2022, alongside more than 18 million vaccine doses administered[6]. In this context, there is limited evidence integrating linked clinical, epidemiological, and vaccination data to specifically examine rehospitalization due to reinfection, particularly in large urban centers, and it remains insufficiently explored to what extent different vaccination statuses, including booster doses, influence the risk of rehospitalization following reinfection".

III. Materials and Methods

Thank you for these detailed and constructive comments. We have carefully revised the Materials and Methods section to improve clarity, transparency, and alignment with reporting standards.

a) Reporting guidelines (STROBE)

Thank you for this suggestion. We have added a statement at the beginning of the Methods section indicating that the study follows the STROBE guidelines, and we have included the corresponding checklist as Supplementary Material, in lines 98-100: “This study was reported in accordance with the STROBE (Strengthening the Reporting of Observational Studies in Epidemiology) guidelines, and the corresponding checklist is provided as Supplementary Material.”

b) Study design (explanatory vs predictive)

We have revised the study design description to explicitly state that this is an explanatory cohort study, clarifying the analytical framework and the interpretation of associations, in line 103: “This is a retrospective explanatory cohort study based on linked administrative databases […]”. Additionally, we reviewed the manuscript to avoid causal language, consistently referring to “factors associated with” the outcome.

c) Exposures, outcome, and follow-up definition

We have clarified the analytical structure of the study by explicitly stating that multiple exposures were evaluated, including demographic, clinical, vaccination-related, and contextual variables, in lines 143-145: “The study evaluated multiple exposures, including demographic, clinical, vaccination-related, and contextual factors, in relation to the risk of rehospitalization due to COVID-19 reinfection.”

We have also redefined the outcome to improve clarity, specifying that it corresponds to time to first rehospitalization due to COVID-19 reinfection, in lines 135-136: “The primary outcome was time to first rehospitalization due to COVID-19 reinfection.”

In addition, we have defined the start and end of follow-up, stating that follow-up begins 91 days after the first hospitalization and continues until reinfection, death (as a competing event), or administrative censoring, in lines 138-141: “Follow-up started 91 days after this date and continued until the first occurrence of: (i) symptom onset for rehospitalization due to reinfection, (ii) death (treated as a competing event), or (iii) administrative censoring at the end of the study period (December 31, 2022), whichever occurred first.” These changes were made to eliminate ambiguity regarding the temporal structure of the cohort.

d) Role of vaccination in the analysis

We have clarified that vaccination status was included as one of several exposures, rather than being treated as the sole exposure of interest. This clarification aligns the analytical framework with the multivariable modeling approach used in the study.

e) Follow-up duration clarification

We have clarified that the study does not assume a fixed one-year follow-up, but instead uses a time-to-event framework. The revised text defines the follow-up period and censoring criteria to avoid misinterpretation.

f) Race/skin color classification

We have clarified that race/skin color classification follows the Brazilian Institute of Geography and Statistics (IBGE) standards, and that the “Black” category includes individuals self-identified as Black or Brown. This clarification was added to ensure proper contextualization of race in the Brazilian setting, in lines 147-149: “race/skin color was classified according to Brazilian Institute of Geography and Statistics (IBGE) categories as White; Black (including individuals self-identified as Black or Brown); and Others (including Yellow, Indigenous, or missing).”

g) Study population and sample size

We have added a statement clarifying that the study used a population-based approach, including all eligible individuals from the linked databases. Accordingly, we explicitly stated that no a priori sample size calculation was performed, in lines 107-109: “The study included all eligible individuals identified in the linked databases; therefore, no a priori sample size calculation was performed”.

h) Statistical analysis transparency (SAMPL)

We have expanded the statistical analysis section to improve transparency, including:

• Normality testing (Kolmogorov–Smirnov test) – line 177: “Categorical variables were expressed as proportions and compared using Pearson’s chi-square test, while continuous variables were tested for normality using the Kolmogorov–Smirnov test and presented as medians and interquartile ranges (IQR) and analyzed using the Kruskal-Wallis test.”

• Explicit definition of effect measures (subdistribution hazard ratios – sHR) – line 186: “This modeling approach allowed for the estimation of subdistribution hazard ratios (sHR) and their respective 95% confidence intervals [...]”

• Description of model assumptions (Schoenfeld residuals) – line 188: “The proportional hazards assumption was assessed using Schoenfeld residuals.”

• Model selection criteria (AIC) – line 189: “Model selection was based on the Akaike Information Criterion (AIC).”

• Statistical significance threshold (p < 0.05) – lines 189-190: “Statistical significance was defined as p < 0.05.”

i) Ethical considerations

We have added a new subsection explicitly stating that patients and the public were not involved in the study design, conduct, or reporting, in lines 201-203: “Patients and the public were not involved in the design, conduct, or reporting of this study.”

IV. Results

Thank you for this important suggestion. We have revised the Results section to improve its organization and clarity.

Specifically, the section now begins with a clear description of the participants’ baseline characteristics, including demographic and clinical variables, as presented in Table 1. We then present the descriptive findings related to rehospitalization rates over time. Finally, we report the results of the main analysis, including the multiple competing risks model assessing factors associated with rehospitalization due to reinfection (Table 2).

As this study did not include secondary outcomes, no additional analyses are presented in this regard.

These revisions can be found in the Results section (pages 9-12).

VI. Discussion

We thank the reviewer for this suggestion. We revised the Discussion section to ensure better alignment with the Methods and Results, improving the clarity and consistency of the interpretation of findings. We also expanded the description of the study limitations, explicitly addressing potential sources of bias, as well as their direction and magnitude, in accordance with recommendations for cohort studies, as presented in lines 317–337. In addition, we strengthened the section by more explicitly incorporating implications for public health policy and clinical practice, particularly regarding the importance of booster vaccination strategies in reducing severe outcomes such as rehospitalization, as described in lines 346–348. Finally, we included recommendations for future research, highlighting the need to incorporate more detailed socioeconomic variables and to assess long-term outcomes in order to further advance the understanding of health inequalities and the lasting impacts of COVID-19, as stated in lines 349–351: “Future studies should incorporate more detailed socioeconomic variables and assess long-term outcomes, to further advance the understanding of health inequalities and the lasting impacts of COVID-19.”

V. Conclusion

Thank you for this valuable suggestion. We have revised the Conclusion section to make it more concise and focused on the main findings of the study, as recommended. Specifically, we removed extended policy-oriented statements and relocated these elements to the Discussion section.

The revised Conclusion now emphasizes the key message regarding the association between vaccination status—particularly booster doses—and the risk of rehospitalization due to reinfection, as well as the role of demographic and clinical factors. Additionally, we included a brief perspective on future research, highlighting the need to further investigate socioeconomic factors and long-term outcomes, as well as to inform preparedness for future respiratory public health emergencies, in lines 355-363: “In summary, this study identified factors associated with SARS-CoV-2 reinfection-related rehospitalization in Rio de Janeiro. A complete vaccination schedule including booster doses was associated with a reduced risk of rehospitalization, while advanced age, multimorbidity, and racial disparities were associated with increased risk. These findings reinforce the role of biological and social determinants in COVID-19 outcomes and highlight the relevance of vaccination status in the context of rehospitalization. Future studies should further explore socioeconomic factors and long-term outcomes to better understand the impacts of the disease, as well as to inform preparedness and response strategies for future respiratory public health emergencies.”

• Minor comments

We thank the reviewer for the suggestions. The manuscript has been revised to ensure full compliance with the author guidelines of PLOS ONE. In this process, we standardized the formatting of references both in the text and in the reference list, revised the Abstract to remove subsections as required by the journal, and eliminated numbering from all sections and subsections. Additionally, the manuscript was aligned with the journal’s formatting template to ensure consistency throughout.

Regarding reporting guidelines, we acknowledge the importance of the RECORD (Reporting of studies Conducted using Observational Routinely-collected health Data) statement for studies based on routinely collected data. Although the study was originally reported in accordance with the STROBE guidelines, we have now reviewed the manuscript to ensure alignment with RECORD recommendations. The Methods section provides a detailed description of the data sources and the record linkage procedures, including the variables used and the criteria applied. Furthermore, the variables and outcomes are clearly defined, and the analytical procedures, including data handling, are explicitly described. The Discussion section was also strengthened to include a more comprehensive assessment of the limitations inherent to administrative databases, addressing potential sources of bias.

Finally, as suggested, we have included all required statements at the end of the manuscript, including data availability, conflicts of interest, and acknowledgments.

• Review Comments to the Author - Major comments

1. We appreciate this observation. The inconsistency has been corrected throughout the manuscript. All effect estimates now consistently report a 39% reduction in risk, corresponding to an sHR of 0.61 derived from the fully adjusted Fine & Gray model. We have also explicitly labeled these estimates as subdistribution hazard ratios (sHR) to avoid ambiguity.

2. We thank the reviewer for this important observation. We acknowledge that the inclusion of unspecified severe acute respiratory illness (SARI) cases in the outcome may introduce potential misclassification by capturing events not necessarily related to SARS-CoV-2 reinfection. However, this decision was made considering the epidemiological context in Brazil during the pandemic, where diagnostic limitations, reporting delays, and under-ascertainment of laboratory confirmation were common, particularly in earlier periods.

To assess the impact of potential misclassification, we conducted a sensitivity analysis restricting the outcome to rehospitalizations with laboratory-confirmed SARS-CoV-2 infection. This analysis showed no statistically significant differences compared to the main analysis, supporting the robustness of our findings. Based on these results, we retained both case definitions to preserve sensitivity in identifying relevant events, in lines 178-180: “A sensitivity analysis comparing rehospitalizations with and without laboratory-confirmed SARS-CoV-2 infection showed no statistically significant differences; therefore, both case definitions were retained.”

3. We thank the reviewer for this important observation. The aim of the study was to assess the association between race/ethnicity, particularly Black race, and the risk of reinfection-related rehospitalization compared with White individuals. We acknowledge that the handling of missing data may introduce bias; however, the proportion of missing data for this variable was low, reducing the likelihood of substantial distortion of the estimates. Nevertheless, we reported the distribution of missing data in the descriptive tables and expanded the discussion to address potential bias related to this variable. The inclusion of a directed acyclic graph (DAG) was not undertaken; instead, we explicitly described the rationale for covariate adjustment based on theoretical knowledge and prior literature.

4. Absence of ICU admission during the initial hospitalization was associated with a lower risk of rehospitalization. This finding likely reflects differences in baseline clinical severity, as ICU admission is generally reserved for more critical cases, whereas general wards manage less severe conditions. Therefore, ICU admission should be interpreted as a proxy for disease severity rather than a causal factor. Additionally, SARS-CoV-2 reinfections have been reported to present with milder clinical manifestations, particularly among individuals who are fully vaccinated with booster doses.

5. We thank the reviewer for the comment. The calculation of annual rehospitalization rates, including the definition of person-time denominators and the procedures used, is already described in detail in the Methods section, in lines 182-183: “Rehospitalization rates were calculated annually as the number of rehospitalizations per year divided by the total person-years of follow-up, multiplied by 100.”

6. We revised the text to clarify that we exclusively used the Fine and Gray model for subdistribution hazards. All estimates were labeled as subdistribution hazard ratios (sHR). We removed the reference to the Schoenfeld test and clarified the procedures used to assess the proportional hazards assumption in the context of competing risks.

7. The Methods section details the modeling of vaccination as a time-dependent variable, including updating vaccination status during follow-up, considering effective immunity 14 days after each dose, and using a counting process data structure. We also clarified the strategies used to avoid immortal time bias.

8. We thank the reviewer for this important comment. The time origin was defined based on the date of symptom onset, as this variable presented greater completeness and consistency across the integrated databases. In contrast, hospital admission and discharge dates showed a high degree of missingness and inconsistencies, which could introduce bias and compromise the reliability of time-to-event estimates.

To ensure internal consistency, both the start of follow-up (91 days after symptom onset) and the definition of the event (time to rehospitalization) were anchored to the same temporal reference (symptom onset). This approach allowed for a standardized and comparable measurement of time at risk across individuals.

We acknowledge that this choice differs from approaches based on hospital admission dates, and this limitation has been explicitly addressed in the Discussion section.

Additionally, this strategy helped minimize potential biases arising from incomplete hospitalization records, ensuring a more robust definition of the risk period.

9. We revised the Methods section to explicitly specify the set of covariates pre-defined based on epidemiological knowledge (age, sex, race/skin color, comorbidities, baseline severity, calendar period/variants, and vaccination). The use of the Akaike Information Criterion (AIC) was retained as a complementary approach and is now described as an exploratory/sensitivity analysis.

10. We thank the reviewer for this important comment. The record linkage process was conducted by the coordinating research team prior to data access, using identifiable information under appropriate ethical and institutional approvals. The dataset made available for this analysis was fully de-identified and did not include linkage keys or individual-level identifiers.

As a result, it was not possible to directly assess linkage performance metrics (such as match rates or false-positive estimates), nor to independently replicate the linkage procedures. Similarly, deduplication of individuals and the definition of hospitalization sequences (index hospitalization and first rehospitalization) were performed beforehand by the coordinating team, following standardized criteria.

11. We acknowledge the potential for residual confounding. We expanded the Discussion section to explicitly address this limitation and included available proxies, such as race/skin color. We also highlight the need for future studies to incorporate more detailed socioeconomic variables and to apply more robust quantitative methods to better control for confounding.

12. We revised the Data Availability section to include more detailed information, specifying the responsible authority (Epidemiological Intelligence Center – CIE), the access criteria, and the institutional contact, in accordance with the journal’s policy.

13. All references have been reviewed and standardized according to the journal’s style. We corrected inconsistencies in volume, issue, and pagination, and included DOIs whenever available.

14. The manuscript structure was thoroughly revised to comply with the journal’s guidelines, removing inappropriate numbering and standardizing headings, subheadings, and figure captions.

15. We included 95% confidence intervals for the main estimates.

16. We standardized the use of the term “rehospitalization” throughout the manuscript, as well as the nomenclature for race/skin color, ensuring textual consistency.

17. We conducted a comprehensive revision of the manuscript, correcting grammatical inconsistencies, punctuation, hyphenation, and clarity of expressions.

18. The manuscript was fully revised to ensure consistency, clarity, and compliance with the journal’s guidelines, including formal and scientific writing aspects.

We sincerely thank the reviewers for their valuable comments and suggestions, which contributed to improving the clarity and quality of the manuscript. We have carefully addressed all major concerns raised. Minor comments and editorial suggestions have already been incorporated into the revised version of the manuscript.

Ranna Kíssia Alves das Neves

Graduate Student, Public Health Epidemiology Program, ENSP/FIOCRUZ

Rio de Janeiro, RJ, Brazil

rannaneves@gmail.com | +55 68 99981-9498

Attachment Submitted filename: Response_to_Reviewers_assinado.pdf

# Peer review: Decision Letter 1

**DOI:** 10.1371/journal.pone.0332281.r003

**Authors:** Mickael Essouma

14 Jun 2026

Dear Dr. Neves,

Thank you for submitting your manuscript to PLOS ONE. After careful consideration, we feel that it has merit but does not fully meet PLOS ONE’s publication criteria as it currently stands. Therefore, we invite you to submit a revised version of the manuscript that addresses the points raised during the review process.

Please submit your revised manuscript by Jul 29 2026 11:59PM. If you will need more time than this to complete your revisions, please reply to this message or contact the journal office at plosone@plos.org. When you're ready to submit your revision, log on to https://www.editorialmanager.com/pone/ and select the 'Submissions Needing Revision' folder to locate your manuscript file.

- A letter that responds to each point raised by the academic editor and reviewer(s). You should upload this letter as a separate file labeled 'Response to Reviewers'.
- A marked-up copy of your manuscript that highlights changes made to the original version. You should upload this as a separate file labeled 'Revised Manuscript with Track Changes'.
- An unmarked version of your revised paper without tracked changes. You should upload this as a separate file labeled 'Manuscript'.

If you would like to make changes to your financial disclosure, please include your updated statement in your cover letter. Guidelines for resubmitting your figure files are available below the reviewer comments at the end of this letter.

If applicable, we recommend that you deposit your laboratory protocols in protocols.io to enhance the reproducibility of your results. Protocols.io assigns your protocol its own identifier (DOI) so that it can be cited independently in the future. For instructions see: https://journals.plos.org/plosone/s/submission-guidelines#loc-laboratory-protocols. Additionally, PLOS ONE offers an option for publishing peer-reviewed Lab Protocol articles, which describe protocols hosted on protocols.io. Read more information on sharing protocols at https://plos.org/protocols?utm_medium=editorial-email&utm_source=authorletters&utm_campaign=protocols.

As the corresponding author, your ORCID iD is verified in the submission system and will appear in the published article. PLOS supports the use of ORCID, and we encourage all coauthors to register for an ORCID iD and use it as well. Please encourage your coauthors to verify their ORCID iD within the submission system before final acceptance, as unverified ORCID iDs will not appear in the published article. *Only*  the individual author can complete the verification step; PLOS staff *cannot*  verify ORCID iDs on behalf of authors.

We look forward to receiving your revised manuscript.

Kind regards,

Mickael Essouma, M. D.

Academic Editor

PLOS One

Journal Requirements:

If the reviewer comments include a recommendation to cite specific previously published works, please review and evaluate these publications to determine whether they are relevant and should be cited. There is no requirement to cite these works unless the editor has indicated otherwise.

Please review your reference list to ensure that it is complete and correct. If you have cited papers that have been retracted, please include the rationale for doing so in the manuscript text, or remove these references and replace them with relevant current references. Any changes to the reference list should be mentioned in the rebuttal letter that accompanies your revised manuscript. If you need to cite a retracted article, indicate the article’s retracted status in the References list and also include a citation and full reference for the retraction notice.

Additional Editor Comments (if provided):

Editor's comments alongside Reviewer's comments

I. Conform to PLOS One author guidelines when formatting the text, including when citing references in the the text. See for example how references are cited in this article: https://journals.plos.org/plosone/article?id=10.1371/journal.pone.0347033

II. The sentence "According to the technical definition of reinfection adopted in Brazil, new symptomatic episodes occurring at least 90 days after the first infection" in lines 82 and 83 is incomplete. Did you wish to write "According to the technical definition adopted in Brazil, COVID-19 reinfection was defined as a new symptomatic episode occurring at least 90 days after the first infection"?

III. I understand that the definition of COVID-19 reinfection in this study was based on the definition adopted in Brazil, but in that definition, did you consider the need to ascertain that the new symptoms emerging 90 days after the first infection occurred after previous biological evidence (eg, through PCR tests) of SARS-COV-2 clearance? Because claiming reinfection means that there was healing from the first infection before the new infection appeared, and healing from COVID-19 infection is best demonstrated by resolution of COVID-19 signs together with biological evidence of SARS-COV-2 clearance. This point should be addressed in the limitations statement of the Discussion section.

IV. Consider avoiding overstatements not directly linked to your findings throughout the Discussion section (eg, statements on social inequality). For instance, the sentence "These disparities reflect the complex intersections between race, socioeconomic status, and healthcare access" in lines 304 and 305 needs to be tempered because you did not demonstrate in this study that Black people had a high risk of reinfection due to low socioeconomic status and limited access to healthcare. You could modify that sentence like this: "These disparities could reflect the complex intersections between race, socioeconomic status, and healthcare access, since Black people generally have a low socioeconomic status and limited access to care in Brazil." Same remark for the sentence in lines 295-297, you could modify that sentence like this: "The simultaneous presence of multiple comorbidities is known to contribute to a proinflammatory state and elevated risk of cytokine storm[44], while immunosenescence hinders infection control[45].". And so on.

V. I suggest further revising the title so that it really aligns with the work done. I suggest this manuscript's title: "Rehospitalization for COVID-19 reinfection in Rio de Janeiro in 2020–2022: effect of vaccination status and sociodemographic and clinical factors". Indeed, you said (in the response letter) that you conducted a time-to-event follow-up, not an annual follow-up. So, I do not see the relevance of the word annual in the manuscript's title. Furthermore, throughout the manuscript, an emphasis is put on the predictors of reinfection, not on the rate of reinfection. Therefore, I also would not keep the word not "rate" in the manuscript's title.

Mickael Essouma, M.D.

[Note: HTML markup is below. Please do not edit.]

Reviewers' comments:

Reviewer's Responses to Questions

**Comments to the Author**

Reviewer #3: All comments have been addressed

Reviewer #4: All comments have been addressed

**********

2. Is the manuscript technically sound, and do the data support the conclusions?

Reviewer #3: Yes

Reviewer #4: (No Response)

**********

3. Has the statistical analysis been performed appropriately and rigorously?

Reviewer #3: Yes

Reviewer #4: (No Response)

**********

4. Have the authors made all data underlying the findings in their manuscript fully available?

The PLOS Data policy

Reviewer #3: Yes

Reviewer #4: (No Response)

**********

5. Is the manuscript presented in an intelligible fashion and written in standard English?

Reviewer #3: Yes

Reviewer #4: (No Response)

**********

Reviewer #3: This is a well-designed population-based cohort study using linked administrative databases to evaluate COVID-19 reinfection-related rehospitalization in Rio de Janeiro. The methodology is appropriate, the statistical analyses are robust, and the findings are clinically and epidemiologically meaningful.

The protective association observed for booster vaccination is convincing and consistent with current evidence, while the identification of racial and multimorbidity-related disparities adds important public health value.

The manuscript is scientifically sound and requires only minor refinements.

Methods

The study addresses a relevant public health question by evaluating rehospitalization due to COVID-19 reinfection and the role of vaccination status in a large urban population. The objectives are clearly stated and the retrospective cohort design is appropriate for the research question

Minor concerns remain:

More information regarding linkage quality metrics (matching accuracy, false matches, linkage sensitivity) would further strengthen confidence in the integrated database.

The grouping of Black and Brown individuals into a single category follows Brazilian epidemiological standards but should continue to be explicitly justified when interpreting inequity-related findings.

Residual socioeconomic confounding remains likely because individual-level socioeconomic variables were unavailable.

Results

The results are presented in a logical sequence, beginning with participant characteristics and progressing to incidence rates and multivariable analyses.

Minor concerns remain:

Additional graphical presentation of cumulative incidence curves by vaccination status could improve interpretability.

Conclusions

The conclusions are supported by the presented results and remain appropriately aligned with the study objectives. The authors avoid overstating causality and consistently frame findings as associations rather than causal effects.

Editorial Suggestions

Consider providing additional details regarding linkage performance metrics.

Expand discussion of possible residual confounding related to socioeconomic status and healthcare access.

Consider adding cumulative incidence plots stratified by vaccination status.

Minor language polishing may further improve readability in several discussion paragraphs.

Reviewer #4: (No Response)

**********

what does this mean?). If published, this will include your full peer review and any attached files.

If you choose “no”, your identity will remain anonymous but your review may still be made public.

**Do you want your identity to be public for this peer review?**  For information about this choice, including consent withdrawal, please see our Privacy Policy

Reviewer #3: No

Reviewer #4: No

**********

[NOTE: If reviewer comments were submitted as an attachment file, they will be attached to this email and accessible via the submission site. Please log into your account, locate the manuscript record, and check for the action link "View Attachments". If this link does not appear, there are no attachment files.]

To ensure your figures meet our technical requirements, please review our figure guidelines: https://journals.plos.org/plosone/s/figures

You may also use PLOS’s free figure tool, NAAS, to help you prepare publication quality figures: https://journals.plos.org/plosone/s/figures#loc-tools-for-figure-preparation.

NAAS will assess whether your figures meet our technical requirements by comparing each figure against our figure specifications.

# Author comment: Author response to Decision Letter 2

**DOI:** 10.1371/journal.pone.0332281.r004

22 Aug 2026

We thank the reviewer for the thoughtful and constructive comments and for recognizing the novelty of our study.

Comment II:

Thank you for this observation. We agree that the original sentence was grammatically incomplete. We have revised the sentence to clearly state the definition of COVID-19 reinfection adopted in the study. This change has been incorporated into the revised manuscript (Lines 82–84), as follows:

“According to the technical definition adopted in Brazil, COVID-19 reinfection was defined as a new symptomatic episode occurring at least 90 days after the first infection”.

Comment III:

Thank you for this important observation. We agree that biological evidence of SARS-CoV-2 clearance between episodes would provide greater certainty in identifying true reinfections. However, in our study, it was not possible to confirm virological clearance for all individuals because longitudinal testing data between episodes were not consistently available in the administrative databases used. Therefore, reinfection was operationally defined using the minimum 90-day interval recommended by the Brazilian Ministry of Health.

In accordance with the reviewer’s suggestion, we have acknowledged the absence of confirmation of virological clearance as a limitation in the Discussion section (Lines 323-325). The following sentence was added:

“In addition, virological clearance between episodes could not be confirmed for all individuals because longitudinal testing data were not consistently available in the databases.”

Comment IV:

Thank you for this important observation. We carefully revised the Discussion section to avoid statements that could imply causal relationships beyond our findings and to more clearly distinguish the associations observed in our study from potential explanations supported by previous literature.

The statements were revised as follows:

Lines 258-260: “This study identified factors associated with SARS-CoV-2 reinfection-related rehospitalization among hospitalized patients in the municipality of Rio de Janeiro, highlighting associations with both biological and sociodemographic factors.”

Lines 263-264: “These findings suggest that both biological and sociodemographic factors may contribute to differences in COVID-19 outcomes.”

Lines 295-297: “It is known that the simultaneous presence of multiple comorbidities contributes to a pro-inflammatory state and elevated risk of cytokine storm[44], while immunosenescence hinders infection control”.

Lines 305-311: “These disparities may reflect the complex intersections between race, socioeconomic status, and healthcare access[53], especially in contexts such as Rio de Janeiro, where 22% of the population live in high social vulnerability areas (favelas)[54]. The higher prevalence of comorbidities in rehospitalized black patients[55] may also contribute to the observed disparities. These findings are consistent with a syndemic perspective on COVID-19, in which social inequities and preexisting vulnerabilities may interact to influence disease outcomes, particularly among socially disadvantaged populations.”

Lines 315-317: “No ICU admission during the initial hospital stay was associated with a lower risk of rehospitalization. One possible explanation is that patients who did not require intensive care had less severe disease during the initial hospitalization”.

Lines 325-327: “In addition, virological clearance between episodes could not be confirmed for all individuals because longitudinal testing data were not consistently available in the databases”.

Comment V:

Thank you for this suggestion. We agree that the terms “annual” and “rates” in the previous title did not adequately reflect the primary focus and analytical approach of the study.

Accordingly, we revised the title to better reflect the study design, outcome, and main explanatory variables. We also chose the term “associations” rather than “effect” to avoid implying causality, considering the observational nature of the study.

The revised title (Lines 1–3) is:

“Rehospitalization due to COVID-19 reinfection in Rio de Janeiro, 2020–2022: associations with vaccination status and sociodemographic and clinical factors.”

Response to Reviewer #3

We thank the reviewer for the careful and constructive evaluation of our manuscript and for the positive assessment of its methodological approach, statistical analysis, and public health relevance. We carefully considered the comments and suggestions provided and reviewed the manuscript accordingly, with particular attention to methodological clarity, interpretation of the findings, and the limitations of the study.

In particular, we agree that the absence of individual-level socioeconomic information may result in residual confounding and should be more explicitly acknowledged. Therefore, we revised the limitations section of the Discussion (Lines 340-342) as follows:

“Finally, the absence of individual-level socioeconomic variables may have resulted in residual socioeconomic confounding and limits a more in-depth analysis of social inequalities.”

We believe that this revision, together with the other language and interpretative refinements made throughout the manuscript, provides a more cautious interpretation of the findings and improves the overall clarity of the Discussion.

Attachment Submitted filename: Response_to_Reviewers_part_2_assinado.pdf

# Editor report: Decision Letter 2

**DOI:** 10.1371/journal.pone.0332281.r005

**Authors:** Mickael Essouma

10 Sep 2026

Rehospitalization due to COVID-19 reinfection in Rio de Janeiro, 2020–2022: associations with vaccination status and sociodemographic and clinical factors

PONE-D-25-45468R2

Dear Dr. Neves,

We’re pleased to inform you that your manuscript has been judged scientifically suitable for publication and will be formally accepted for publication once it meets all outstanding technical requirements. Congratulations!!!

Within one week, you’ll receive an e-mail detailing the required amendments. When these have been addressed, you’ll receive a formal acceptance letter and your manuscript will be scheduled for publication.

An invoice will be generated when your article is formally accepted. Please note, if your institution has a publishing partnership with PLOS and your article meets the relevant criteria, all or part of your publication costs will be covered. Please make sure your user information is up-to-date by logging into Editorial Manager at Editorial Manager® and clicking the ‘Update My Information' link at the top of the page. For questions related to billing, please contact billing support.

If your institution or institutions have a press office, please notify them about your upcoming paper to help maximize its impact. If they’ll be preparing press materials, please inform our press team as soon as possible -- no later than 48 hours after receiving the formal acceptance. Your manuscript will remain under strict press embargo until 2 pm Eastern Time on the date of publication. For more information, please contact onepress@plos.org.

Kind regards,

Mickael Essouma, M. D.

Academic Editor

PLOS One

Additional Editor Comments (optional):

Reviewers' comments:

# Editor report: Acceptance letter

**DOI:** 10.1371/journal.pone.0332281.r006

**Authors:** Mickael Essouma

PONE-D-25-45468R2

PLOS One

Dear Dr. Neves,

I'm pleased to inform you that your manuscript has been deemed suitable for publication in PLOS One. Congratulations! Your manuscript is now being handed over to our production team.

At this stage, our production department will prepare your paper for publication. This includes ensuring the following:

* All references, tables, and figures are properly cited

* All relevant supporting information is included in the manuscript submission,

* There are no issues that prevent the paper from being properly typeset

You will receive further instructions from the production team, including instructions on how to review your proof when it is ready. Please keep in mind that we are working through a large volume of accepted articles, so please give us a few days to review your paper and let you know the next and final steps.

Lastly, if your institution or institutions have a press office, please let them know about your upcoming paper now to help maximize its impact. If they'll be preparing press materials, please inform our press team within the next 48 hours. Your manuscript will remain under strict press embargo until 2 pm Eastern Time on the date of publication. For more information, please contact onepress@plos.org.

You will receive an invoice from PLOS for your publication fee after your manuscript has reached the completed accept phase. If you receive an email requesting payment before acceptance or for any other service, this may be a phishing scheme. Learn how to identify phishing emails and protect your accounts at https://explore.plos.org/phishing.

If we can help with anything else, please email us at customercare@plos.org.

Thank you for submitting your work to PLOS One and supporting open access.

Kind regards,

PLOS One Editorial Office Staff

on behalf of

Dr. Mickael Essouma

Academic Editor

PLOS One
